# Emergence of linguistic laws in human voice

**DOI:** 10.1038/srep43862

**Published:** 2017-03-08

**Authors:** Iván González Torre, Bartolo Luque, Lucas Lacasa, Jordi Luque, Antoni Hernández-Fernández

**Affiliations:** 1 Department of Applied Mathematics and Statistics, EIAE, Technical University of Madrid, Plaza Cardenal Cisneros, 28040, Madrid, Spain; 2 School of Mathematical Sciences, Queen Mary University of London, Mile End Road, E14NS, London, UK; 3 Telefonica Research, Edificio Telefonica-Diagonal 00, Barcelona, Spain; 4 Complexity and Quantitative Linguistics Lab, Laboratory for Relational Algorithmics, Complexity and Learning (LARCA), Institut de Ciències de l’Educació, Universitat Politècnica de Catalunya, Barcelona, Spain

## Abstract

Linguistic laws constitute one of the quantitative cornerstones of modern cognitive sciences and have been routinely investigated in written corpora, or in the *equivalent* transcription of oral corpora. This means that inferences of statistical patterns of language in acoustics are biased by the arbitrary, language-dependent segmentation of the signal, and virtually precludes the possibility of making comparative studies between human voice and other animal communication systems. Here we bridge this gap by proposing a method that allows to measure such patterns in acoustic signals of arbitrary origin, without needs to have access to the language corpus underneath. The method has been applied to sixteen different human languages, recovering successfully some well-known laws of human communication at timescales even below the phoneme and finding yet another link between complexity and criticality in a biological system. These methods further pave the way for new comparative studies in animal communication or the analysis of signals of unknown code.

The main objective of quantitative linguistics is to explore the emergence of statistical patterns (often called linguistic laws) in language and general communication systems (see refs 1,2 for a review). The most celebrated of such regularities is Zipf’s law describing the uneven abundance of word frequencies^3^,^4^. This law presents many variations in human language^5^,^6^,^7^,^8^ but also shows ubiquity^9^ in many linguistic scales^10^, has been claimed to be universal^11^,^12^ and has consequences for syntax and symbolic reference^13^. On the other hand Heaps’ law, also called Herdan’s law^14^,^15^ states that the vocabulary of a text grows allometrically with the text length^16^,^17^, and is mathematically connected with Zipf’s law^18^,^19^,^20^, the scaling exponent being dependent on both Zipf law and the vocabulary size. Finally the Zipf’s Law of abbreviation (or brevity law for short) is the statistical tendency of more frequent elements in communication systems to be shorter or smaller^4^,^21^ and has been recently claimed as an universal trend derived from fundamental principles of information processing^22^. As such, this statistical regularity holds also phonetically^23^,^24^ and implies that the higher the frequency of a word, the shorter its length or duration, probably caused by a principle of compression^25^,^26^, although this is a general pattern that can change depending on other acoustical factors like noise^27^, pressure to communicate at long distances calls^28^ or communicative efficiency^4^ and energetic constraints^29^.

Linguistic laws extend beyond written language and have been shown to hold for different biological data^7^,^30^. According to some authors^31^, the presence of scaling laws in communication is indicative of the existence of processes taking place across different cognitive scales. Interpreting linguistic laws as scaling laws which emerged in communication systems^32^,^33^ actually opens the door for speculating on the existence of underlying scale-invariant physical laws operating underneath^34^. Of course, in order to explore the presence or absence of such patterns one needs to directly study the acoustic corpus, i.e. human voice, as every linguistic phenomenon or candidate for language law can be camouflaged or diluted after the transcription process. Notwithstanding the deep relevance of linguistic laws reported in written texts, we still wonder which of these laws found in written corpora are related or derive from more fundamental properties of the acoustics of language -and are thus candidates for full-fledged linguistic laws-, or emerge as an artifact of scripture codification.

## Linguistic Laws in Acoustics?

Acoustic communication is fully determined by three physical magnitudes extracted from the signals: frequency, energy and time^35^,^36^. The configuration space induced by these magnitudes results from an intrinsic evolutionary relationship between the production and perception of sound systems^37^ that further shapes the range of hearing and producing sounds for a variety of life forms^35^,^38^. Since animals use their acoustic abilities both to monitor their environment and to communicate we should expect that natural selection has in some sense optimized these sensorial capabilities, but, despite the great differences that evolution has involved for different animals that communicate acoustically, there are many similarities between their mechanisms of sound production and hearing^39^. Focusing on primates, traditionally language has been distinguished from vocalizations of nonhuman primates only at a qualitative, semantic level^40^. Interestingly, it is well known that children use statistical cues to segment the input^41^,^42^,^43^ and probably share with non-human primates some of these mechanisms, albeit with some differences^44^. The discovery of these statistical learning abilities has boosted a new approach to the study of language^45^,^46^ and suggests that statistical learning of language could be based on patterns or more generally linguistic laws^22^: research on language acquisition shows that higher frequency facilitates learning, and Zipf’s law tell us that vocabulary learning is easier than expected a priori given the skewness of the distribution, for instance. We advance that, as will be shown below, these patterns are already present in the physical sound waves produced by human voice even at levels below those generally considered linguistically significant, i.e., below the phoneme timescale.

Empirical evidence of robust linguistic laws holding in written texts across different human languages has been reported many times (see refs 2, 7 and 19 and references therein), and it has been shown that these laws are not fully observed in random texts^48^. Studies with oral corpus are however much less abundant, and they systematically imply a transcription of the acoustical waves into words in the case of human speech or some ill-defined analog of words in the case of animal communication, as the main segments to analyze^49^,^50^. A few current efforts take a different road and consider other possible written units such as lemmas^51^ or compare written and oral production for some linguistic patterns^52^,^53^, in general showing that frequencies of elements in written corpora can be taken as a rough estimate for their frequency in spoken language^54^.

All in all, the exploration of linguistic laws in oral corpora is scarce. In fact, all linguistic studies in oral and written corpora are influenced by our segmentation decisions and our definition of ‘word’, intimately biased by an inherently anthropocentric perspective and, of course, by our linguistic tradition. The idea that speech is produced like writing, as a linear sequence of vowels and consonants may indeed be a relic of our scripture technology. As a matter of fact, it is well-known in linguistics that both vowels and consonants are produced linearly but also depend on their surrounding elements: this is the traditional and well-studied concept of coarticulation^55^. The boundaries between acoustical elements are therefore difficult to identify if we are not native speakers of a language, and that’s yet a crucial problem of phonotactics and speech segmentation and recognition^56^ with differences across languages^57^.

Precisely because of this, classical signal segmentation based on the concept of “word” inherited from writing has lead some researchers to search how to transform artificially written corpora into phoneme or syllabic chains with different objectives^58^, at the time it involves two major problems in communication studies, namely (i) the impossibility of performing fully objective comparative studies between human and non-human signals^59^, where signals could be physical events, behaviours or structures to which receivers respond^60^. This problem leads researchers sometimes to manually segment acoustic signals guided only by their expertise, and prevents to explore signals of unknown origin including for instance the search for possible extraterrestrial intelligence^61^. And (ii) a rather arbitrary definition of the units of study guided by ortographic conventions already produces non-negligible epistemological problems at the core of Linguistics^62^,^63^.

In this work we explore the acoustic analog of classical linguistic laws in the statistics of speech waveform from extensive real databases^64^ that for the first time extend all the way into the intraphoneme range (*t* < 10^−2^ s)^65^. In order to do so in a systematic way, in what follows we will present a methodology that enables the direct linguistic analysis of any acoustical waveform without needs to invoke to *ad hoc* codes or assume any particular formal communication system underneath. This method only makes use of physical magnitudes of the signal such as energy or time duration which therefore allow for a non-ambiguous definition of our units. Speech and voice are indeed physical phenomena and as such in this work we interpret them as a concatenation of events of energy release, and propose a mathematically well-defined way for segmenting in time this orchestrated suite of energy release avalanches^47^. We will show clear evidence of robust Zipf, Heaps and brevity laws emerging in this context and speculate that this might be due to the fact that human voice seems to be operating close to a critical state, hence finding an example of a biological system that, driven by evolution, has linked complexity and criticality. We expect that this methodology can open a fresh line of research in communication systems where a direct exploration of underlying statistical patterns in acoustic signals is possible without needs to predetermine any of the aforementioned non-physical concepts, and hope that this will allow researchers to develop comparative studies between human language and other acoustical communication systems or even to unravel whether if a generic signal shares these patterns^2^.

## Data

For this work we have used two databases: the main one is a TV broadcast speech database named KALAKA-2^64^, and additionally a complementary database named LRE has been used to confirm results on a larger set of languages. Originally designed for language recognition evaluation purposes, the former consists of wide-band TV broadcast speech recordings (4 hours per language sampled using 2 bytes at a rate of 16 kHz) ranging six different languages: Basque, Catalan, Galician, Spanish, Portuguese and English and encompassing both planned and spontaneous speech throughout diverse environmental conditions, such as studio or outside journalist reports but excluding telephonic channels. The second one complements KALAKA with an additional set of 12 languages, taken from the NIST Language Recognition Evaluation (LRE′96) corpus and including Japanese, Vietnamese, Mandarin, Korean, Arabic, Hindi or Tamil to cite some (see SI for details).

## The Method

**Figure 1:**
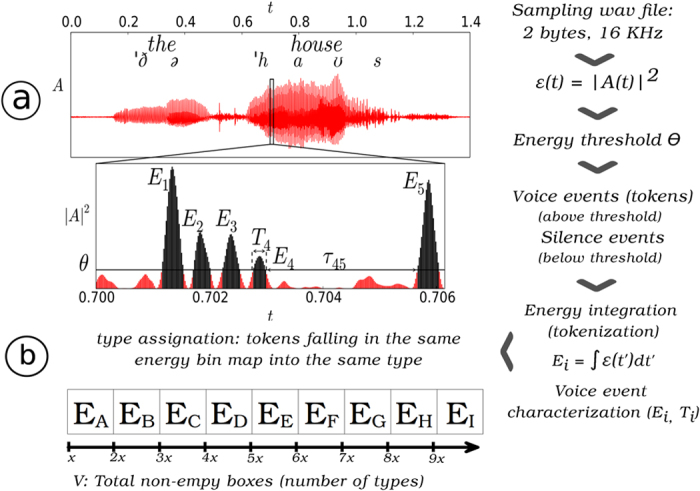
This figure illustrates the methodology to extract a sequence of types from an acoustic signal. Waveform series *A(t*) are sampled at 16 KhZ from the system. In (a) we consider an excerpt from *A(t*) that coincides with an individual articulating the words “the house”. In the middle panel we plot the instantaneous energy per unit time *ε(t*) = |*A*^2^|(*t*) from an excerpt of the top panel. The energy threshold Θ, defined as the instantaneous energy level for which a fixed percentage of the entire data remains above-threshold, helps us to unambiguously define a token or voice event (a subsequence of time stamps for which *ε(t*) > Θ) from silence events of duration *τ*^47^. The energy released *E*_*i*_ in voice event *i* (where *i* ∈ ℕ) is computed from the integration of the instantaneous energy over the duration of that event *T*_*i*_ (dark area in the figure denotes the energy released in a given voice event). Subsequently, by performing a linear binning tokens are classified into bins that we call types (in the plot, *E*_*A*_, *E*_*B*_,… are different bins). The vocabulary *V* agglutinates those types that appear at least once.

The objects under study, speech waveforms or otherwise any generic acoustic signal, are fully described by an amplitude time series *A(t*) (see Fig. 1 for an illustration of the method). In order to unambiguously extract a sequence of elements -the equivalent to words and phrases- from such signal without the need to perform *ad hoc* segmentation, we start by considering the semi-definite positive magnitude *ε(t*) = |*A(t*)|^2^ which, dropping irrelevant constants, has physical units of energy per time (SI for additional details on speech waveform statistics). By defining an energy threshold Θ in this signal^47^ we will unambiguously separate voice events (above threshold) from silence events (below threshold). More concretely, Θ is defined as a relative percentage of the signal and its actual value in energy units depends on the signal variability range: for example Θ = 80% means that 20% of the data falls under this energy level. It has been shown that Θ decimates the signal similarly to a real-space Renormalization Group (RG) transformation^47^,^66^, in such a way that increasing Θ induces a flow in RG space. Systems operating close to a critical state lie in an unstable fixed point of this RG flow and its associated signal statistics are therefore shown to be threshold-invariant. Now, Θ not only works as an energy threshold that filters out background or environmental noise (noise filtering being a key aspect that species have learned to perform^27^,^67^) but, as previously stated, enable us to unambiguously define what we call a *token* or voice event, that is, a sequence of consecutive measurements *ε(t*) > Θ, from a silence event of duration *τ*. Each token is in turn characterized by a duple (*E*_*v*_, *T*_*v*_) where *T*_*v*_ is the duration of the event and *E*_*v*_ corresponds to the total energy released during that event 
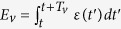
 obtained summing up the instantaneous energy over the duration of the event. Accordingly, the signal is effectively transformed to an ordered sequence of tokens {(*E*_*v*_(*i*), *T*_*v*_(*i*))}, each of these being separated by silence events of highly heterogeneous durations *τ* which, incidentally, are known to be power law distributed^47^. Finally, by linearly binning the scale of integrated energies we can assign an energy label (the bin) to each token, hence mapping the initial acoustic signal into a symbolic sequence of fundamental units which we call *types*. Note that two tokens whose integrated energy fall within the range of the same energy bin are mapped to the same type even if their duration can be different, so in principle several tokens could map into the same type (see SI for a table of Type/Token ratios). The linear binning of the energy scale has a bin size b = 0.01, in such a way that the first bin agglutinates tokens with energies between E0 and E0 + b, the second bin between E0 + b and E0 + 2b, and so on (note that results reported later will be robust against changes in b). The set of bins can be understood as an abstraction of a universal language vocabulary, and accordingly some bins might be empty and in general each bin will occur with uneven frequencies. As such, types can be understood as acoustically-based universal abstractions of a fundamental unit, an abstract version of words or phonemes that appear intertwined in a signal with characteristic patterns.

To synthesize, with this methodology we are able to map an arbitrary acoustic signal into a sequence of types separated by silence events (Fig. 1). Standard linguistic laws can then be directly explored in acoustic signals without needs to have an *a priori* knowledge neither of the signal code nor of the adequate segmentation process or the particular syntax of the language underlying the signal. This protocol is thus independent of the communication system and can be used to make unbiased comparisons across different systems and signals. Needless to say, results could in principle depend on the particular value of Θ, as this scans the signals at different energy thresholds. However human voice has been recently shown^47^ to be invariant under changes in Θ -an evidence of self-organized criticality (SOC)^68^ in this system- and, accordingly, parameter-free laws can be extracted using a proper collapse theory as it will be shown in the results section. Also, in order to guarantee that the emergence of linguistic laws is only due to the structure and correlations of the signal and not due to the process of symbolization we will compare the results obtained from speech signals to properly defined null models which randomize the signal *ε(t*). These null models thus maintain the marginal instantaneous energy distribution and remove any other correlation structure, yielding non-Gaussian white noise with a fat-tailed marginal distribution.

It should be highlighted that, according to the abovementioned protocol, the types extracted do not share in principle a direct relation with real words or with any other linguistic units, but only a formal one as these types constitute a base of fundamental acoustic units of any oral communication system. As a matter of fact, we consider this possibility to be unlikely, as our basic units of study, while ranging several scales, are in most of the cases smaller than the typical timescales of words. Further work should explore to what extent there is a connection between tokens, or sequences of them, and phonemes, words or other linguistic units. Notwithstanding, this a priori lack of matching is indeed required and desired if one aims at developing a method which is universally applicable in communication signals of arbitrary origin, not necessarily oral communication based on words. Such is the case, for instance, in the realm of comparative analysis across different species. In any case, note that our method relies on retrieving voice events via energy thresholding. As many of the detected events typically belong to the intraphoneme range, one could argue that the method focus on the rich composition of voice and silence events within a given linguistic unit (phonemes or words).

Despite not being the purpose of this work, this method may be seen as an unsupervised Voice Activity Detection (VAD) algorithm. It is well known that VAD algorithms based on temporal domain analysis or on energy features are highly affected by noise but note that VAD systems are typically optimized through reference transcriptions, manually annotated at the word level^69^,^70^. The purpose of our approach is however not to perform word or phoneme level segmentation, but rather to explore organisational patterns of language-independent acoustic units taking place at different scales in oral communication, and quantify them via classical linguistic analysis. That being said, assessing how informative are those cues in (unsupervised) word segmentation or speech recognition tasks^71^,^72^,^73^ is an interesting question that will deserve further investigation.

## Results

### Gutenberg-Richter law

**Figure 2:**
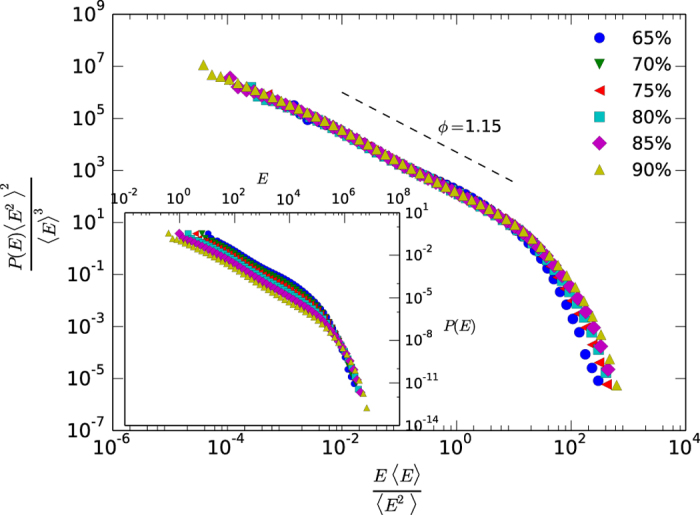
Log-log plot of the collapsed shape (see the text) and threshold-independent energy release distribution *P(E*) in the case of Spanish language (KALAKA database) for several thresholds, after logarithmic binning. (Inset panel) Non-collapsed distribution, for different thresholds.

The energy *E* released during voice events is a direct measure of the vocal fold response function under air pressure perturbations, and its distribution *P*_Θ_(*E*) could in principle depend both on the threshold Θ and on the language under study. In the inset of Fig. 2 we observe that *P*_Θ_(*E*) is power law distributed over about six decases, saturated by an exponential cut-off. This distribution has been interpreted before as the analogue of a Gutenberg-Richter law in voice, as the precise shape of energy release fluctuations during voice production parallel those occurring in earthquakes^47^. As increasing Θ induces a flow in RG space, systems which lie close to a critical point (unstable fixed point in RG space) show scale invariance under Θ and hence the distributions can be collapsed into a Θ-independent shape, thereby eliminating the trivial dependence on Θ. This has been shown to be the case for human voice and accordingly (technical details can be found in the SI) we can express the collapsed energy distribution as *P(E*) = *E*^−*ϕ*^
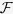
(*E*/*E*_*ξ*_) for 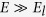, where *E*_*l*_ is the lower limit beyond which this law is fulfilled, 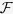 is a scaling function and the relevant variable is *ϕ*, the scaling exponent. In order to collapse every curve *P*_Θ_(*E*) the theory predicts to rescale *E* → *E*〈*E*〉/〈*E*^2^〉, *P*_Θ_(*E*) → *P*_Θ_〈*E*^2^〉^2^/〈*E*〉^3^. In the outset of Fig. 2 we show the result of this analysis for the case of Spanish language, where we find that *ϕ* ≈ 1.15, this exponent being approximately language-independent (see Table 1 for other languages and SI for additional details). Interestingly, these exponents are compatible with those found in rainfall, another natural system that has been shown to be compatible with SOC dynamics^74^, and cannot be explained by simple null models^47^. While the precise values of the exponents are not extremely relevant, one should take these with caution as thresholding is known to introduce in some cases finite size biases in the form of a second scaling region for short sizes which is only removed when the series is long enough^75^. In what follows we explore the emergence of classical linguistic laws in these acoustic signals.

### Zipf’s Law

The illustrious George Kingsley Zipf formulated a statistical observation which is popularly known as Zipf’s law^76^. In its original formulation^3^, it establishes that in a sizable sample of language the number of different words (vocabulary) *N(n*) which occur exactly *n* times decays as *N(n*)~*n*^−*ζ*^, where the exponent *ζ* varies from text to text^77^ but is usually close to 2. An alternative and perhaps more common formulation of this law^4^ is defined in terms of the rank, such that if words are ranked in decreasing order by their frequency of appearance then the number of occurrences of words with a given rank *r* goes like *n(r*)~*r*^−*z*^, where it is easy to see that both exponents are related via 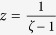 and thus *z* approximates to 1^16^,^78^. Here for convenience we make use of the former and explore *N*_Θ_(*n*) applied to the statistics of types. Interestingly, while Zipf’s (and Heaps’s) laws were originally proposed to quantify order in the word arrangement on written texts, these laws indeed describe the arrangement of informational units that go beyond words in texts (see for instance^7^,^11^) and there exist several theoretical justifications for the presence of these laws which do not require resorting to linguistics but to more fundamental concepts such as principles of compression in information theory^12^,^26^ which don’t necessarily require the units to adhere to any particular type of communication.

As a matter of fact, a mathematical relation between the energy release statistics and Zipf’s law can be found. First, note that the frequency of type *r* is proportional to


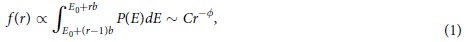


where the asymptotic approximation is easily found expanding 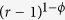 up to second order. Now, since *P(E)* is monotonically decreasing, then there is a formal equivalence between type *r* and rank *r*: the most frequent type corresponds to type ‘1’ and thus has rank 1, the second most frequent type is ‘2’, and so on: type with label *r* will have rank *r*, as frequencies of types are naturally ordered in a monotonically decreasing way by construction. Therefore, the frequency of rank *r* is proportional to eq. 1. Accordingly 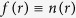 so we predict *z* ≈ *ϕ* and thus 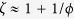.We now consider experimental results.

**Figure 3:**
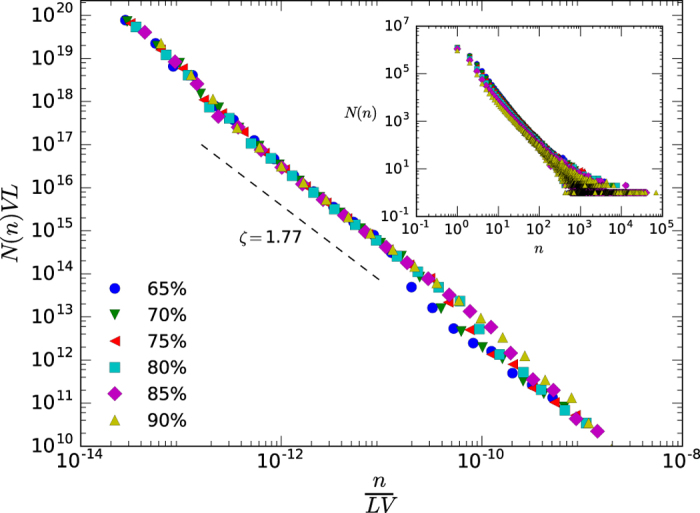
Log-log plot of Zipf’s law for the case of Basque language (KALAKA database, see SI for equivalent results in other languages), for different thresholds Θ. The inset panel shows the raw, threshold-dependent distributions and in the outer panel Zipf’s law has been collapsed (see the main text for details) using a logarithmic binning. The scaling exponent here is *ζ* ≈ 1.77.

Again *N*_Θ_(*N*) could in principle depend on the threshold but assuming that the signal complies with the scale-invariance mentioned above, one can collapse all threshold-dependent curves into a universal shape and thus remove any dependence on this parameter by rescaling *n* → *n*/*LV, N*_Θ_(*n*) → *N*_Θ_(*n)VL* where *V* is the total number of different types present in the signal and *L* as the total number of tokens (see SI for technical details). Results are shown in the case of Basque language in Fig. 3, where a clear threshold-independent decaying power law emerges with a scaling exponent *ζ* ≈ 1.77. Analogous results with compatible exponents for other languages can be found in SI and Table 1. The relation between exponents ζ and ϕ is on good agreement with our theoretical prediction. Null models systematically deviate from these results, and neither display the characteristic power law decay nor any invariance under variation of the energy threshold (SI).

### Heaps’ law

**Figure 4:**
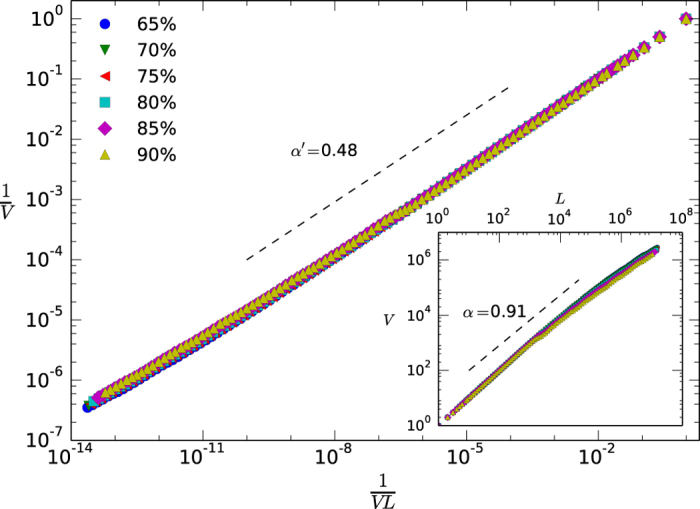
Log-log plot of the Heaps’ law for the Portuguese sample (KALAKA database) and several thresholds. In the inner panel we show how the number of different tokens (V) increases sublinearly with the size of the series (L), where the slope can be estimated properly for about three decades. In the main panel we show the collapses curves following^16^, where the new scaling exponent *α*′ ≈ 0.48 is related with the original (see the text) and leads to *α* ≈ 0.91. Results for other languages are found in Table 1.

Together with Zipf’s law and connected mathematically (see ref. 2 and references therein), the second classical linguistic law is Heaps’ law, the sublinear growth of the number of different words *V* in a text with text size *L* (measured in total number of words): *V *~ *L*^*α*^, *α *< 1^14^,^79^ (a constant rate for appearance of new words leads to *α* = 1). Here the vocabulary *V* is defined as the total number of different types that appear in the signal, whereas *L* is defined as the total number of tokens found for a given threshold. Results are shown for a specific language in Fig. 4 (see SI for the rest). In the outset panel we present the collapsed, threshold-independent curves, where again we find a scaling law with an effective exponent *α*′ related to the original exponent *α*′ = *α*/(1 + *α*). In this case equivalent computation on the null model yield a Heaps law with the trivial exponent *α*  ≈ 1 (SI).

These results are quantitatively consistent with previous results on written texts^16^. In particular, several authors^18^ point out that, at least asymptotically, the relation *ζ *= 1 + *α* holds with good approximation, and this is on reasonably good agreement with our findings in human voice as well. Interestingly, a recent work^80^ has found that, as opposed to Indoeuropean (alphabetically based) languages, Zipf’s law breaks down and Heaps’ law reduces to the trivial case for written texts in Chinese, Japanese, Korean and other logosyllabic languages. Applying our methodology in the database of such logosyllabic languages (Japanese, Mandarin, Korean) our results are at odds with^80^ as we still find evidence of Zipf and Heaps laws holding for these languages in terms of the energetic voice fluctuations. We conclude that those differences found when written texts are analyzed do not arise when we study directly human voice.

### Brevity Law

**Figure 5:**
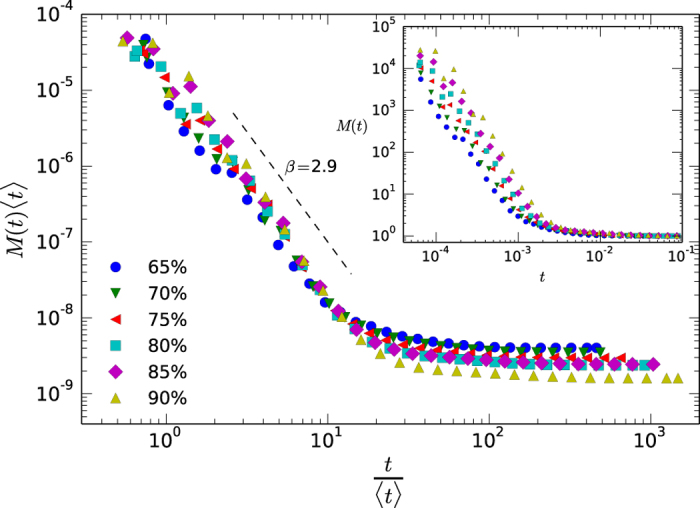
Log-log plot of the Brevity law in the case of English (KALAKA database), for several thresholds. In the inner panel we plot, for different thresholds, the histogram *M*_Θ_(*t*) that describes the relative frequency of a type of mean duration *t*. In every case we find a monotonically decreasing curve which yields a brevity law. In the outset panel we present the collapsed, threshold-independent curve *M(t*), that evidences an initial power law decay with an exponent *β* ≈ 2.9.

The tendency of more frequent words to be shorter^3^,^4^,^21^ can be generalized as the tendency of more frequent elements to be shorter or smaller, and its origin has been suggested to be related to optimization and information compression arguments in connection with other linguistic laws^81^. In acoustics, spontaneous speech indeed tends to obey this law after text segmentation^82^, and has been found also in other non-human primates^28^,^81^. Here we can test brevity law in essentially two different ways. First, note that voice events (tokens) map into types according to the logarithmic binning of their associated energy, hence voice events with different duration might yield the same type as previously noted. Thus for each type we can compute its mean duration *t* averaging over all voice events that fall within that type, and then plot the histogram *M*_Θ_(*t*) that describes frequency of each type versus its mean duration. Brevity law would require *M*_Θ_(*t*) to be a monotonically decreasing function. These results are shown for a particular language in log-log scales in Fig. 5, finding initially a power law decaying relation which is indicative of a brevity law (results are again found to be language independent, see SI for additional results). The inset provide the threshold-dependent distributions and the outset panel provides the collapsed, threshold-independent shape *M(t*) (see Table 1 for scaling exponents). Again in this case results in null models deviate from such behavior (SI) and are clearly different from the random typing^12^. Alternatively, one can also directly observe the duration frequency at the level of voice events, finding similar results (see SI).

## Discussion

In this work we have explored the equivalent of linguistic laws directly in acoustic signals. We have found that human voice manifests the analog of classical linguistic laws found in written texts (Zipf’s law, Heaps’ law and the brevity law or law of abbreviation). These laws are found to be invariant under variation of the energy threshold Θ, and can be collapsed under universal functions accordingly. As Θ is the only free parameter of the method, this invariance determines that the results are not afflicted by ambiguities associated to arbitrarily defining unit boundaries. Results are robust across a list of 16 different languages (indoeuropean and non-indoeuropean, including some logosyllabic), across timescales (extending all the way into the intraphoneme range, where no cognitive effects operate, and invariant under energy threshold variation) and across conversational modes (one or more speakers, both planned and spontaneous speech throughout diverse environment conditions, such as studio, outside journalist reports, and telephonic channel). Interestingly, an equivalent analysis performed on null models defined by randomizing the signal *ε(t*) (yielding white noise with the same instantaneous energy distribution of the original signal) fail to reproduce this phenomenology (SI). The concrete range of exponents found for both Zipf and Heaps laws are compatible between each other and somewhat similar -but not identical- to the typical ones observed in the literature for written texts^2^,^7^,^76^,^80^, whereas to the best of our knowledge this is the first observation of scaling behavior with a clear exponent in the case of brevity law in speech. Actually, our finding of a power law in brevity law differs from the case of random typing where a power law doesn’t conform^12^.

The specific and complex alternation of air stops (silences) intertwined with voice production are at the core of the microscopic voice fluctuations. During voice production, acoustic communication is governed by the so-called biphasic cycle (breath and glottal cycle, see ref. 83 for a review) that together with some other acoustic considerations (pitch period, voice onset time, the relation between duration, stress and syllabic structure^82^) determines the microscopic structure of human voice, including silence stops. However, these timescales are in general very large: as previously stated, this current study focus and scans voice properties even at intraphonemic timescales, where the statistical laws of language emerge directly from the physical magnitudes that govern acoustic communication. Our results therefore open the possibility of speculating whether the fact that these laws have been found in upper levels of human communication might be a result of a scaling process and a byproduct of the physics rather than derived from the choice of the typical units of study on the analysis of written corpus (phonemes, syllabus, words, …), like differences between analysis of Indoeuropean and logosyllabic languages demonstrates^80^. As a matter of fact, in a previous work human voice has been framed within self-organized criticality (SOC), speculating that the fractal structure of lungs drives human voice close to a critical state^47^, this mechanism being ultimately responsible for the microscopic self-similar fluctuations of the signal. This constitutes a new example of the emergence of SOC in a physiological system, different in principle from the classical one found in neuronal activity^84^. In the same line, note that the set of critical exponents characterizing the SOC nature of human voice have been shown to be very similar to the ones found in another SOC system: rainfall^74^. The celebrated theory of critical phenomena thoroughly explains that many physical processes, when poised close to critical point, essentially reduce to the same phenomenon despite of having different physical origins, and can be described by a set of critical exponents that classifies the core phenomenon in universality classes. As rainfall and voice production share the same critical exponent for energy release, this suggests that in essence a similar mechanism could be triggering both types of threshold-like dynamics.

In any case, one could thus speculate that the emergence of linguistic scaling laws is just a consequence of the presence of SOC, what in turn would support the physical origin of linguistic laws. From an evolutionary viewpoint, under this latter perspective human voice, understood as a communication system which has been optimized under evolutionary pressures, would constitute an example where complexity (described in terms of robust linguistic laws) emerges when a system is driven close to criticality, something reminiscent of the celebrated edge of chaos hypothesis^85^. Furthermore, under this interpretation the onset of complexity in language (as reported by linguistic laws in written texts) could be a direct byproduct of similar patterns already emanating at purely physiological grounds (the physics of energy fluctuation in voice production): in this respect the role played by cognitive processes would be unclear, as the timescales involved in the physiological mechanisms of voice production (intraphonemic) are typically shorter than those associated to cognitive ones. More succinctly, the classical explanation for the origin of scaling laws in language in terms of scaling phenomena in cognitive processes could be challenged by this new evidence. Certainly, we are not claiming that statistical laws of language are only a consequence of human physiology, among other reasons because the clearest evidence leads us to consider the presence of these laws in texts^2^,^7^, but the plausible causal link between scaling laws in acoustics and the analogous ones at the language level is suggestive and should be studied in depth.

On more practical grounds, the method used and proposed here also addresses the longstanding problem of signal segmentation. It has been acknowledged that there is no such thing as a ‘correct’ segmentation strategy^86^. In written corpus white space is usually taken as an easy marker of the separation between words, however this is far from evident in continuous speech where separation between words or concepts is technologically harder to detect, conceptually vague and probably ill-defined. Few exceptions that used oral corpus for animal communication still require to define ad hoc segmentation algorithms^28^, or manual segmentation strategies which usually give an arbitrary or overestimated segmenting times or windows^81^, what might even raise epistemological questions. As such, this segmentation problem unfortunately has prevented wider, comparative studies in areas such as animal communication or the search for signs of possible extraterrestrial intelligence from radio signals (in this line only few proposals have been made^61^). By varying the energy threshold the method presented here automatically partitions and symbolizes the signal at various energy scales, providing a recipe to establish an automatic, general and systematic way of segmenting and thus enabling comparison of across acoustic signals of arbitrary origin for which we may lack the syntax, code or exact size of its constituents.

To round off, we hope that this work paves the way for new research avenues in comparative studies. Open questions that deserve further work abound; just to name a few: in the light of this new method, what can we say about the acoustic structure in other animal communication systems? Can we find evidence of universal traits in communication that do not depend on a particular species but are only physically and physiologically constrained, or on the other hand are linguistic universals a myth^87^? How these laws evolve with aging^6^,^88^? Are they affected by cognitive or pulmonary diseases? What is the precise relation between SOC and linguistic laws in this context? And in particular, can we find mathematical evidence of a minimal, analytically tractable SOC model that produce these patterns? These and other questions are interesting avenues for future work.

## Additional Information

**How to cite this article**: Torre, I. G. *et al*. Emergence of linguistic laws in human voice. *Sci. Rep.* **7**, 43862; doi: 10.1038/srep43862 (2017).

**Publisher's note:** Springer Nature remains neutral with regard to jurisdictional claims in published maps and institutional affiliations.

## Supplementary Material

Supplementary Information

## Figures and Tables

**Table 1:** Summary of scaling exponents associated to the energy release distribution (*ϕ*), Zipf’s law (*ζ*), Heaps’ law (*α*) and Brevity law (*β*) for six different languages (KALAKA database).

Exponent	*ϕ*	*ζ*	*α*	*β*
Basque	1.13 ± 0.04	1.77 ± 0.14	0.90 ± 0.03	3.1 ± 0.3
Catalan	1.17 ± 0.05	1.89 ± 0.14	0.92 ± 0.03	2.8 ± 0.4
English	1.16 ± 0.05	1.85 ± 0.14	0.91 ± 0.01	2.9 ± 0.3
Galician	1.18 ± 0.04	1.80 ± 0.14	0.89 ± 0.03	2.9 ± 0.4
Portuguese	1.16 ± 0.05	1.77 ± 0.14	0.91 ± 0.01	3.0 ± 0.3
Spanish	1.15 ± 0.04	1.79 ± 0.14	0.91 ± 0.03	2.8 ± 0.4

Power law fits are performed using maximum likelihood estimation (MLE) following Clauset^89^ and goodness-of-fit test and confidence interval are based on 2500 Kolmogorov-Smirnov (KS) tests. The p-value for the evaluation is defined as the fraction of synthetic sample distributions with a KS distance to the best-fit power law that is larger than the KS distance between the empirical distribution and its best-fit power law model. In all cases, the bootstrap p-value of the Kolmogorov-Smirnov test is greater than 0.99, meaning that 99% out of 2500 times the synthetic sampled distribution is closer to the empirical data, hence implying that the power law hypothesis can not be rejected. Exponents associated to energy release are compatible with those found in rainfall^74^. Results are compatible with the hypothesis of language-independence.
