# Supplementary material for: Emergence of linguistic laws in human voice
Source: Sci Rep. 2017 Mar 8;7:43862. doi: 10.1038/srep43862 (PMC5341060; doi:10.1038/srep43862)
Supplement: Supplementary Information [file srep43862-s1.pdf]

Emergence of linguistic laws in human voice (Supplementary Information)

Iván González Torre[a], Bartolo Luque[a,b], Lucas Lacasa[b,*], Jordi Luque[c], and Antoni Hernández-Fernández[d]

[a]Department of Applied Mathematics and Statistics, EIAE, Technical University of Madrid, Spain
[b]School of Mathematical Sciences, Queen Mary University of London, Mile End Road, London E14NS, UK
[c]Telefonica Research, Edificio Telefonica-Diagonal 00, Barcelona, Spain
[d]Complexity and Quantitative Linguistics Lab, Laboratory for Relational Algorithmics, Complexity and Learning, Institut de Ciències de l'Educació, Universitat Politècnica de Catalunya, Barcelona, Catalonia, Spain

Abstract

This is the supplementary material for the paper entitled "Emergence of linguistic laws in human voice". Here we present additional analytical derivations and data analysis.

1 Experimental setup and databases

Two different audio corpus has been used, yielding information on a total of sixteen languages that span a wide range of the language groups and diversity: Basque, Catalan, Egyptian, English, Farsi, French, Galician, German, Hindi, Japanese, Korean, Mandarin, Portuguese, Spanish, Tamil and Vietnamese. In what follows we provide concrete information of these corpus.

- A TV broadcast speech database named KALAKA-2 (Luis Javier Rodríguez-Fuentes, Mikel Peñagarikano, Amparo Varona and Mireia Díez and Germán Bordel, KALAKA-2: a TV Broadcast Speech Database for the Recognition of Iberian Languages in Clean and Noisy Environments, *LREC*, 99–105, (2012)) was employed for analyzing language-dependent speech. It was originally designed for language recognition evaluation purposes and consists of wide-band TV broadcast speech recordings (roughly 4 hours per language) featuring 6 different languages: Basque, Catalan, Galician, Spanish, Portuguese and English. It includes both planned and spontaneous speech throughout diverse environment conditions, such as studio or outside journalist reports but excluding telephonic channel. Therefore audio excerpts may contain voices from several speakers but only a single language. Recordings were done directly from Digital TV with CD quality (16 bit / 44.1 kHz / stereo) through a home connection to cable TV, by means of a Roland Edirol R-09 ultra-light digital audio recorder (`http://www.roland.com/products/en/R-09`). Audio signals were downsampled to 16 kHz, left and right channels being averaged into one single channel, by means of SoX (`http://sox.sourceforge.net`). This way, storage requirements were reduced in a factor of 5.51, while keeping an acceptable (wide-band) quality commonly used in speech processing applications. The resulting signals were stored using 16-bits per

sample, at 16kHz and PCM format. KALAKA-2 recordings were made at three different times: October-November 2008 (English), April-May 2010 (English and Portuguese) and August-September 2010 (Basque, Catalan, Galician and Spanish). Only segments extracted based on high SNR (clean speech or low-level background noise) were used in this work.

- Additionally, a second dataset was analyzed. The language excerpts were extracted from the development audio corpus employed by NIST, the National Institute of Standards and Technology, in the Language Recognition Evaluation (LRE) in 1996. The speech signals correspond to one side of a 4-wire telephone conversation and are represented as standard 8-bit 8 kHz mu-law digital telephone data. In this work audio samples were converted into 2-bytes PCM digital format previously to further processing. The conversations were drawn primarily (but not necessarily exclusively) from LDC's CallFriend corpus (CallFriend Corpus," Linguistic Data Consortium (LDC), 1996. The audio segments belonging to the 30 seconds development condition of LRE96' (lid96d1 in `http://www.itl.nist.gov/iad/mig//tests/lang/1996/LRE96EvalPlan.pdf`) were concatenated in order to expand the language samples leading to audio samples lasting two and four hours (in the case of Spanish, English and Mandarin which two different dialects were pooled together). It is worth to note that, by doing so, different speakers from several conversations, but speaking the same language, and different microphone setups and background conditions were concatenated in the same audio file accounting for one specific language.
  The target languages in NIST Language Recognition Evaluation (LRE) 1996 corpus include English (general american and southern american), Arabic (Egyptian), Canadian French, Mandarin (from mainland China and from Taiwan), German, Hindi, Japanese, Spanish (Caribbean and Highland), Korean, Tamil and Vietnamese. Note that such languages spawn several grapheme-to-phoneme (letter-to-sound) relationships, ranging from logographic languages, with no relationship at all like as Chinesse or Korean, or phonographic ones both segmental and syllabic, like Spanish with almost a 1 to 1 correspondence or Arabic where no short vowels are written.

2 Waveform amplitude and power statistics

The estimated probability density function (marginal distribution) of a speech signal in the time domain has been a matter of investigation since 1950s. While several candidates have included the gamma, Laplacian, Gaussian or generalized Gaussian distributions [2, 5, 4, 1], recent evidence [3] supports that this distribution is indeed better described by the Laplacian distribution only during voice activity while for the samples compounds by a mixture of silences and voices events, the gamma distribution fits better. The amplitude of the signal A should thus be distributed as

$$P(A) = \frac{1}{2h!a^{h+1}}|A|^h e^{-\frac{1}{a}|A|},$$

where a and h are constants.

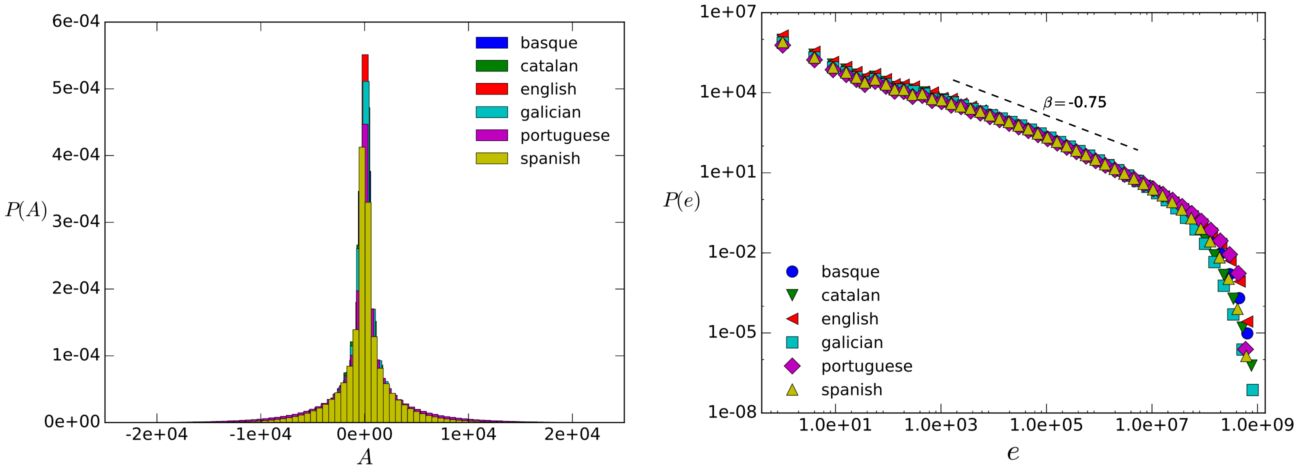

Figure 1: (Left panel) Empirical amplitude distribution $P(A)$ from the speech signals of the set of six languages considered in this work. (Right panel) Log-log plot of the instantaneous energy (power) distribution $P(\epsilon)$ for all languages studied in this work.

The variable $A^2 \equiv \epsilon$ has units of power (energy per unit time) or in other words this is an instantaneous energy. The signal has therefore an instantaneous energy whose distribution can easily found by taking $A = \sqrt{\epsilon}$ such that

$$\frac{dA}{d\epsilon} = \frac{1}{2\sqrt{\epsilon}}.$$

The marginal distribution of ϵ is therefore obtained via a simple change of variable

$$P(\epsilon) = P(A(\epsilon))\frac{dA}{d\epsilon} = \frac{1}{4h!a^{h+1}}\epsilon^{\frac{h-1}{2}}\exp(-\sqrt{\epsilon}/a) \tag{1}$$

that is, a power law distribution with an exponential cutoff. The slope for the power law distribution depends on parameter h. Using a $h = -1/2$ according to [3], the slope expected for the power law distribution will be $-3/4$. The exponential decay depends in general on the properties of each signal via the constant a. In figure 1 we have confirmed the accuracy of this prediction. Note that in practice the probability of finding events with large ϵ is very low. In order to improve the statistics, from now on and all over this work we perform the standard logarithmic binning of the data. For each bin we estimate the frequency of the event and then divide it by the size of that bin.

3 Additional details and discussion on the methods

In figure 2 we plot, for each signal, the number of voice events that are obtained via thresholding for different thresholds. In every case we find that the number of voice events seems to be maximized approximately around a range of values $\theta \in [60\%, 75\%]$ which is an interval where environmental noise has been properly removed but the decimation of the signal is still reasonably low to allow sufficient statistics.

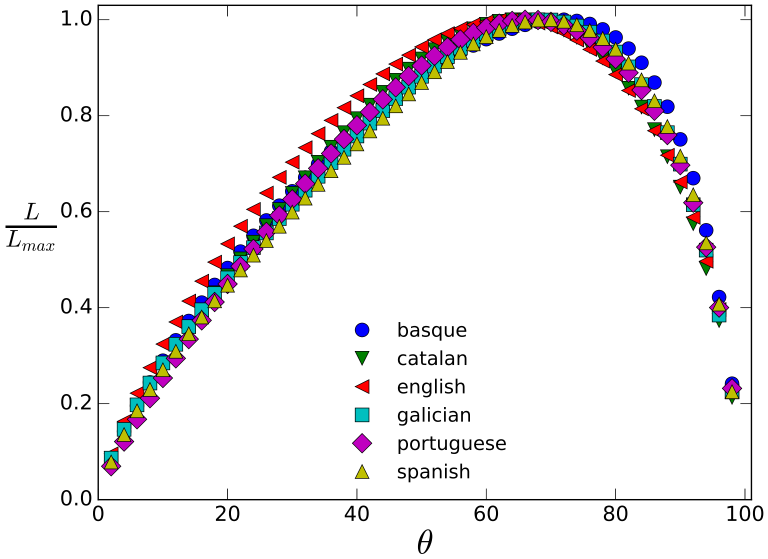

Figure 2: Relative number of voice events L (normalized) as a function of Θ. The voice event taxonomy maximizes for all languages for a range of values of $\theta \in [60\%, 75\%]$

Note that alternatively, the existence of a threshold can be interpreted as an indicator of the distance between the transmitter and the receiver of an acoustic communication signal. Accordingly, the distance between both should be a monotonic function of the threshold θ. The existence of an optimum threshold (as related to a maximum in the number of voice events) might have an evolutive explanation, as in principle the complexity of the communication signal depends on the richness and heterogeneity of the constituents (voice events).

3.1 Collapse theory for self-organized critical (SOC) processes

This section is partially adapted from (F. Font-Clos, G. Boleda, A. Corral, A scaling law beyond Zipf's law and its relation to Heaps' law. New Journal of Physics 15, 093033 (2013)).

In SOC models, the moments of the avalanche size distribution scale with system size L like

$$\langle E^k \rangle \propto L^{D(1+k-\tau)} \text{ for } k > \tau - 1, \tag{2}$$

defining the exponent D, sometimes called the avalanche dimension, and the exponent τ, which we call the avalanche size exponent. Equation 2 is consistent with probability density functions $P(E)$ of the form

$$P(E) = E^{-\tau} \mathcal{F}(E/E_\xi) \text{ for } E > E_l \tag{3}$$

where $E_\xi = L^D$, and the scaling function $\mathcal{F}(E/E_\xi)$ falls off very fast for large arguments, $E/E_\xi > 1$, and is constant for small arguments, $E/E_\xi \ll 1$, down to a lower cutoff, $E = E_l$, where non-universal microscopic effects (*e.g.* discreteness of the system) become important.
Assuming that our observations are generated by a system evidencing SOC, and that a significant part of the observed avalanche sizes are in the region $E_l < E \ll E_\xi$, we expect to find a range of scales where the power law

$$P(E) = \mathcal{F}(0) E^{-\tau} \tag{4}$$

holds. From equation 2 and $E_\xi = L^D$ we have:

$$\langle E \rangle \propto E_\xi^{2-\tau} \text{ and } \langle E^2 \rangle \propto E_\xi^{3-\tau},$$

then

$$E_\xi^{-1} \propto \langle E \rangle / \langle E^2 \rangle \text{ and } E_\xi^{\tau} \propto \langle E^2 \rangle^2 / \langle E \rangle^3,$$

provided $E_l \ll E_\xi$. Hence, to account for changes in effective system sizes the E-axis can be rescaled to: $E\langle E \rangle / \langle E^2 \rangle$. This collapses the loci of the large-scale cutoffs. Plotting $P(E)E_\xi^\tau \propto P(E)\langle E^2 \rangle^2 / \langle E \rangle^3$ against this rescaled variable produces the scaling function $\mathcal{F}(E/(aE_\xi))$, where a is the proportionality constant relating E_ξ to the moment ratio.

Collapse methodology for Zipf and Heap's laws. Zipf's law is obtained directly by counting the number of repetitions, i.e., the absolute frequency n of all tokens in the voice signal and assigning increasing ranks $r = 1, 2, \ldots,$ to decreasing frequencies. When a power-law relation

$$n \propto \frac{1}{r^\beta}$$

holds for a large enough range, with the exponent β more or less close to 1, Zipf's law is considered to be fullfilled (with $\propto$ denoting proportionality). An equivalent formulation of the law is obtained in terms of the probability distribution of the frequency n, such that it plays the role of a random variable, for which a power-law distribution

$$D(n) \propto \frac{1}{n^\gamma},$$

should hold, with $\gamma = 1 + 1/\beta$ (taking values close to 2) and $D(n)$ as the probability mass function of n (or the probability density of n, in a continuous approximation).

Somehow related to Zipf's law is Heaps' law. If we define L as the total number of voice events (tokens) in the signal and V_L as the number of different types in the signal, Heaps' law states that the vocabulary V_L grows as a function of L following

$$V_L \propto L^\alpha,$$

with the exponent α smaller than one.
Let us come back to the rank-frequency relation, in which the absolute frequency n of each type is a function of its rank r. Defining $x \equiv \frac{n}{V_L L}$ and inverting the relationship, we can write

$$r = G_L(x).$$

Note that here we are not assuming a power-law relationship between r and x, just a generic function G_L, which may depend on the text length L. We just need one assumption, which is the independence of the function G_L with respect to L; so our ansatz is:

$$r = G\Big(\frac{n}{V_L L}\Big). \tag{5}$$

This turns out to be a scaling law, with $G(x)$ a scaling function. Now let us introduce the survival function or complementary cumulative distribution function $S_L(n)$ of the absolute frequency, defined in a signal of L tokens as $S_L(n) = \text{Prob}[\text{frequency} \geq n]$. Note that, estimating from empirical data, $S_L(n)$ turns out to be essentially the rank, but divided by the total number of ranks, V_L, i.e., $S_L(n) = r/V_L$. Therefore, using our ansatz for r we get

$$S_L(n) = \frac{G\Big(\frac{n}{V_L L}\Big)}{V_L}.$$

Within a continuous approximation the probability mass function of n, $D_L(n) = \text{Prob}[\text{frequency} = n]$, can be obtained from the derivative of $S_L(n)$,

$$D_L(n) = -\frac{\partial S_L(n)}{\partial n} = \frac{g\left(\frac{n}{V_L L}\right)}{L V_L^2}, \tag{6}$$

where g is minus the derivative of G, i.e., $g(x) = -G'(x)$. If one does not trust the continuous approximation, one can write $D_L(n) = S_L(n) - S_L(n+1)$ and perform a Taylor expansion, for which the result is the same, but with $g(x) \simeq -G'(x)$. In this way, we obtain simple forms for $S_L(n)$ and $D_L(n)$, which are analogous to standard scaling laws, except for the fact that we have not specified how V_L changes with L.
In this work, however, we have represented Zipf law using absolute frequency vs number of tokens, namely

$$N(n) \propto \frac{1}{n^\gamma}$$

Note that

$$N(n) = D_L(n) V_L = \frac{g\left(\frac{n}{V_L L}\right)}{L V_L}$$

Thus, the validity of the proposed scaling law can be checked by performing a very simple rescaled plot, displaying $N(n) L V_L$ versus $n/(V_L L)$.A resulting data collapse support the independence of the scaling function with respect to L.

In the case of Heap's law now, in the continuous approximation,

$$\langle n \rangle = \int_1^\infty S_L(n')dn' = \int_1^\infty \int_n^\infty D_L(n')dn'dn = \int_1^\infty \int_n^\infty \frac{g\left(\frac{n'}{V_L L}\right)}{L V_L^2} dn'dn$$

With the change of variable $x = n'/(V_L L)$ we have

$$\langle n \rangle = \int_1^\infty \int_{\frac{n}{V_L L}}^\infty \frac{g(x)}{V_L} dxdn = \int_1^\infty \frac{G\left(\frac{n}{V_L L}\right)}{V_L} dn$$

With another change of variable $y = n/(V_L L)$, we have

$$\langle n \rangle = \int_{\frac{1}{V_L L}}^\infty L G(y)dy = L H\left(\frac{1}{V_L L}\right)$$

Finally, since $L = \langle n \rangle V_L$ we obtain

$$\frac{1}{V_L} = H\left(\frac{1}{V_L L}\right)$$

4 Type-Token ratio

The Type-Token Ratio (TTR) is perhaps the most frequently used lexical richness measure in Linguistics. TTR calculates the number of different words (types) over the total number of words (tokens) in a corpus. In table 1 we summarize the TTR found for each language and each threshold considered. TTR gives here only an estimate of the diversity of voice events obtained with our automatic segmentation method. Our results are clearly different from the typical in the analysis of lexical richness of oral corpus, perhaps because this is highly dependent on the length of the corpora (the longer a text, the lower the TTR).

	65%	70%	75%	80%	85%	90%
Basque	0.18	0.15	0.16	0.15	0.14	0.14
Catalan	0.16	0.15	0.15	0.15	0.15	0.15
English	0.19	0.17	0.16	0.15	0.15	0.14
Galician	0.14	0.13	0.13	0.13	0.13	0.13
Portuguese	0.19	0.18	0.17	0.16	0.16	0.15
Spanish	0.17	0.16	0.15	0.15	0.14	0.14

Table 1: Type-Token Ratio (TTR) for each language and Threshold (θ)

5 Fitting power laws: additional detais

We have followed the battery of methods proposed by Clauset et al., which consists of using Maximum Likelihood Estimation (MLE) together with Kolmogorov-SMirnov test to fit the best power law model. First, there were some particular cases that merit explicit mention:

- In the case of the instantaneous energy, one cannot actually make use of MLE as the slope of the power law is less than one in this particular case. So we have employed a logarithmic binning and further made a linear regression on a log-log scale. The procedure is similar to MLE, namely the slope is estimated for different low cut-offs x_{min}, such that we select the one which yields a smaller KS distance between the cumulative distribution function (CDF) of the original data and the fitted CDF, that is looking at the point of maximum discrepancy between the cumulative distribution function (CDF) of the original data and the fitted CDF.
- For Heaps's law, as this is a function rather than a distribution, the KS method is not really applicable. In such a case we apply the methodology assuming that this is a discrete distribution.
- For Brevity's law, the scaling range is rather noisy and the MLE does not converge properly, so we apply the procedure in the first item to work out the fitting.

For the rest, goodness-of-fit test and confidence intervals are based on 2500 Kolmogorov-Smirnov (KS) tests. P-value for the evaluation is defined as the fraction of synthetic sample distributions with a KS distance to the best-fit power law that is larger than the KS distance between the empirical distribution and its best-fit power law model. In all cases, the bootstrap p-value of the Kolmogorov-Smirnov test is greater than 0.99, meaning that 99% out 2500 times the synthetic sampled distribution is closer to the empirical data, hence implying that the power law hypothesis can not be rejected.

6 Energy release distribution: additional plots

In this section we plot the energy release distributions for all languages considered in this study. Results are qualitatively similar across languages and exponents are compatible with a language-independent process. (Note that as the KALAKA database is larger than the LRE database, the curves for the corresponding languages associated to the KALAKA case are smoother and the fitting exponents have less error than the ones associated to the LRE database). We also report the analogous distribution obtained from the null model of the spanish database, consisting of reshufling the instantaneous energy signal $\epsilon(t)$. We don't observe a good collapse in this latter case.

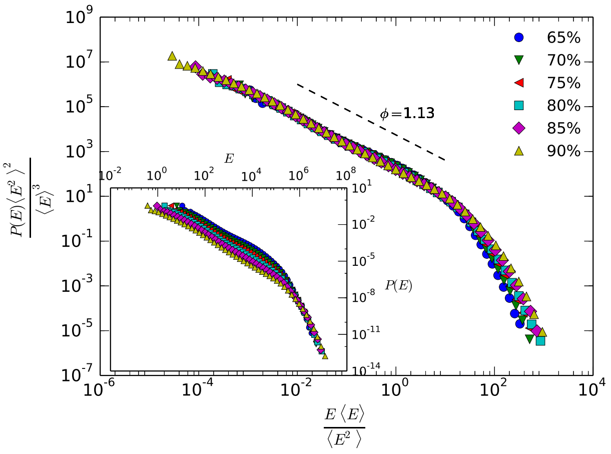

(a) Basque language

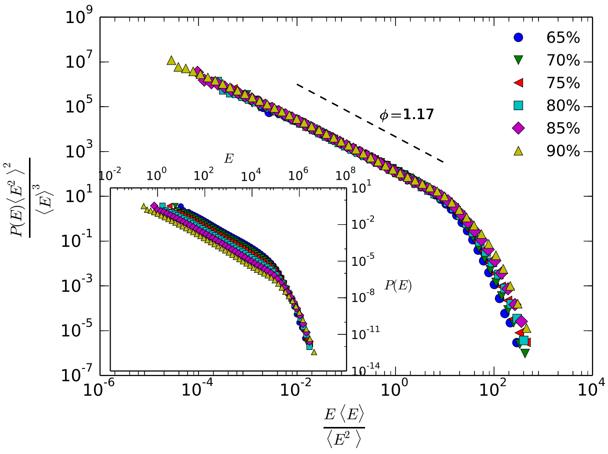

(b) Catalan language

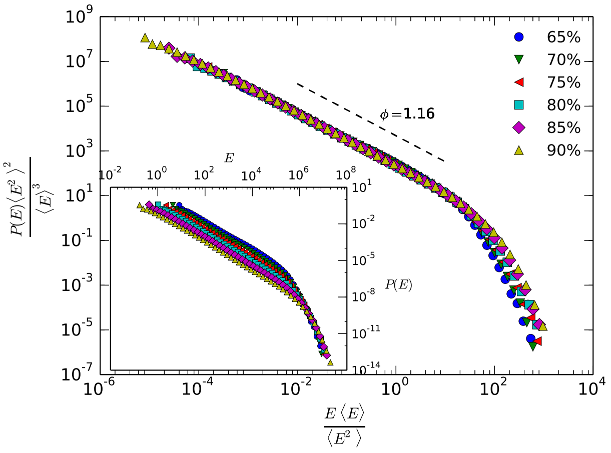

(c) English language

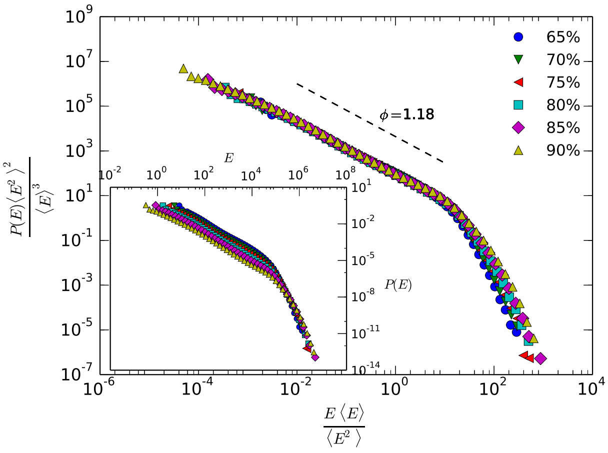

(d) Galician language

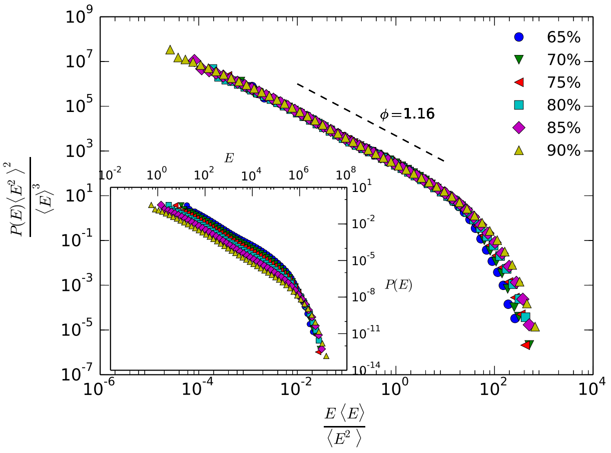

(e) Portuguese language

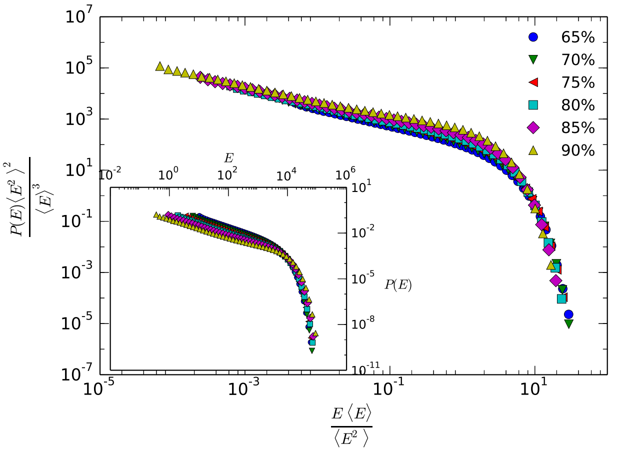

(f) Spanish null model

Figure 3: **Energy distributions (KALAKA dataset).** Panels a to e correspond to each language of the Kalaka database. (Outset panels) Log-log plot of the collapsed threshold-independent energy release distribution $P(E)$ in the case of several thresholds, after logarithmic binning. (Inset panels) Non-collapsed distribution, for different thresholds.Panel f corresponds to the spanish null model wehere energy release distribution is not fulfilled (note that in the null model distributions do not properly collapse)

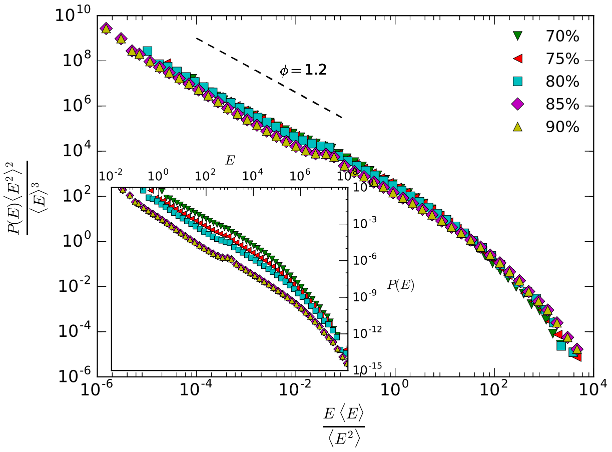

(a) Arabic language

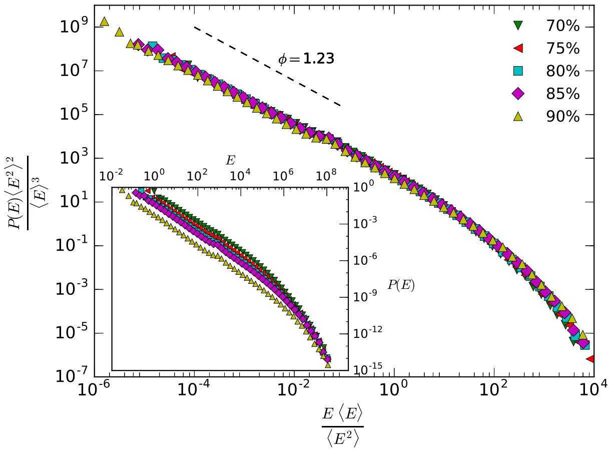

(b) English language

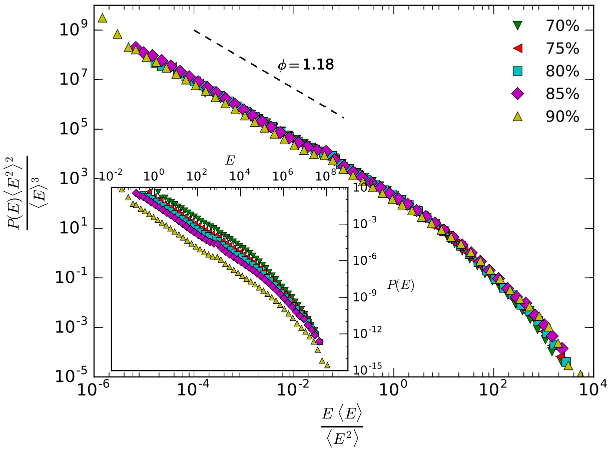

(c) Farsi language

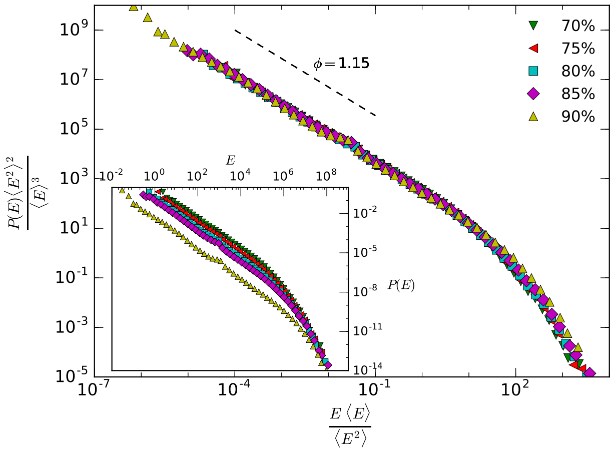

(d) French language

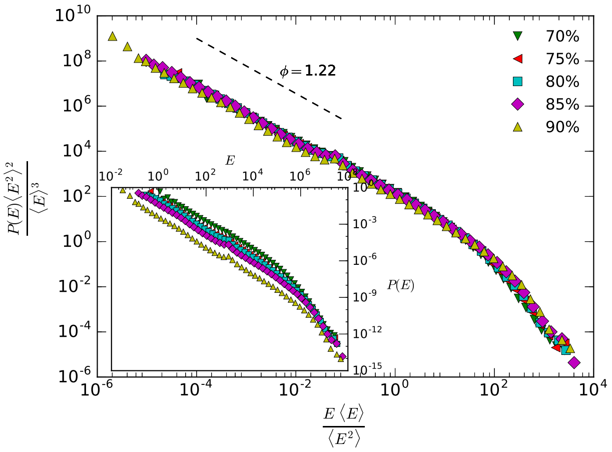

(e) German language

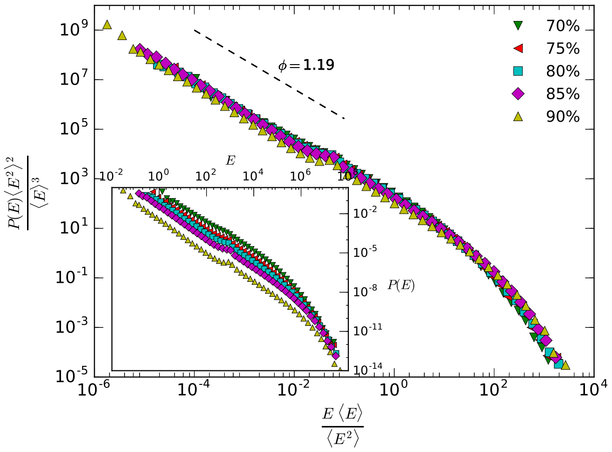

(f) Hindi language

Figure 4: **Energy distribution (LRE database).** Each panel corresponds to one different language of LRE speech database (more languages of this database in Figure 5). (Outer panels) Log-log plot of the collapsed and threshold-independent energy release distribution $P(E)$ in the case of several thresholds, after logarithmic binning. (Inset panels) Non-collapsed distribution, for different thresholds.

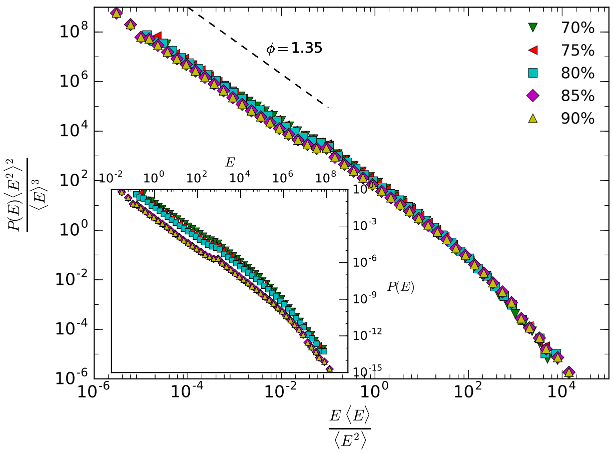

(a) Japanese language

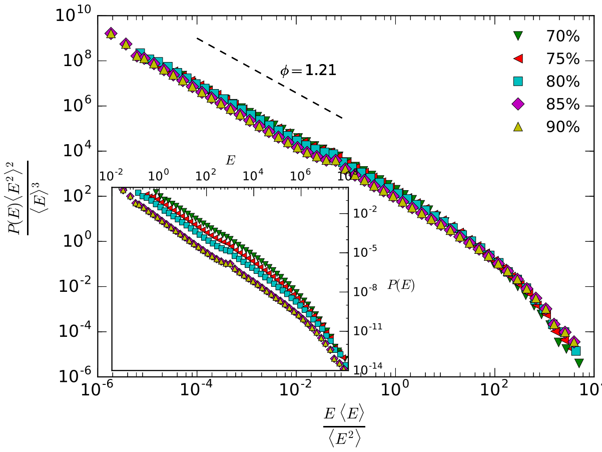

(b) Korean language

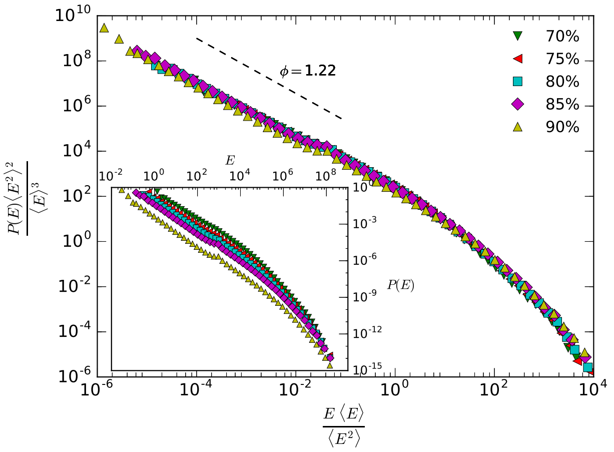

(c) Mandarin language

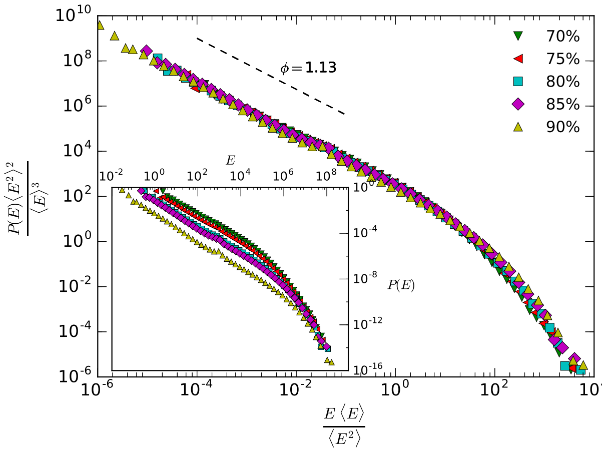

(d) Spanish language

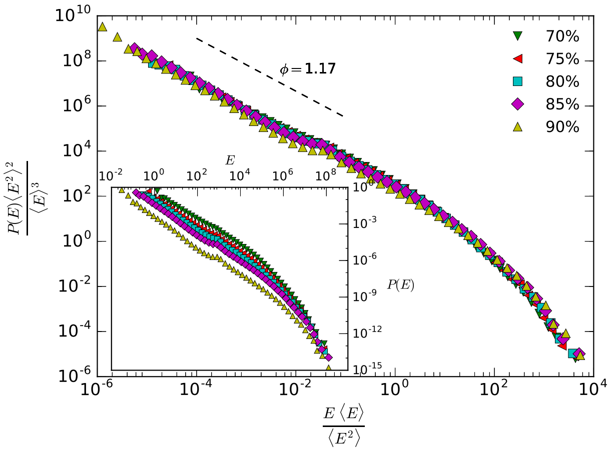

(e) Tamil language

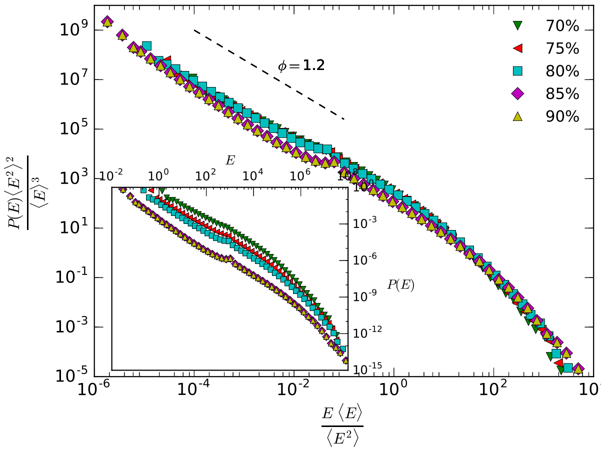

(f) Vietnamese language

Figure 5: **Energy distribution (LRE database).** Each panel corresponds to one different language of LRE speech database. (Outer panels) Log-log plot of the collapsed and threshold-independent energy release distribution $P(E)$ in the case of several thresholds, after logarithmic binning. (Inset panels) Non-collapsed distribution, for different thresholds.

7 Zipf's law: additional plots

In this section we plot the Zipf laws for all languages considered in this study. Results are qualitatively similar across languages and exponents are compatible with a language-independent process. We also report the analogous law obtained from the null model of the basque database, consisting of reshufling the instantaneous energy signal $\epsilon(t)$. We don't observe a good collapse in this latter case (results are equivalent for the null models generated for all other languages). (Note again that as the KALAKA database is larger than the NIST database, the curves for the corresponding languages associated to the KALAKA case are smoother and the fitting exponents have less error than the ones associated to the LRE database).

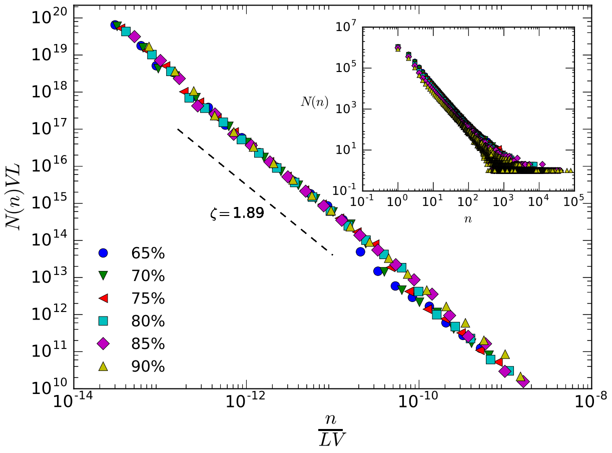

(a) Catalan language

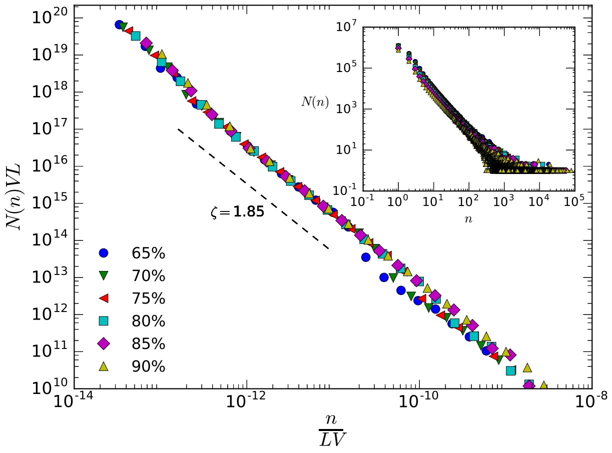

(b) English language

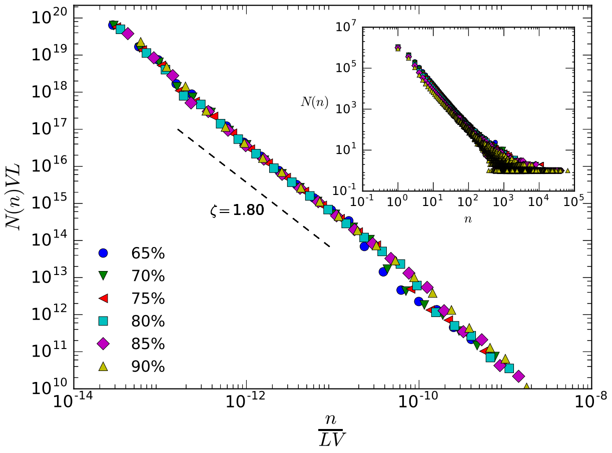

(c) Galician language

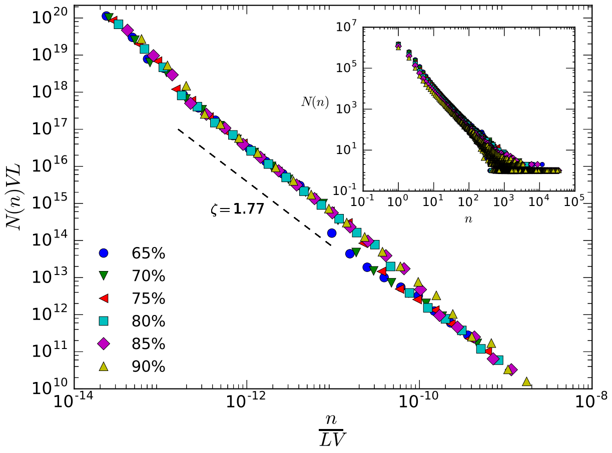

(d) Portuguese language

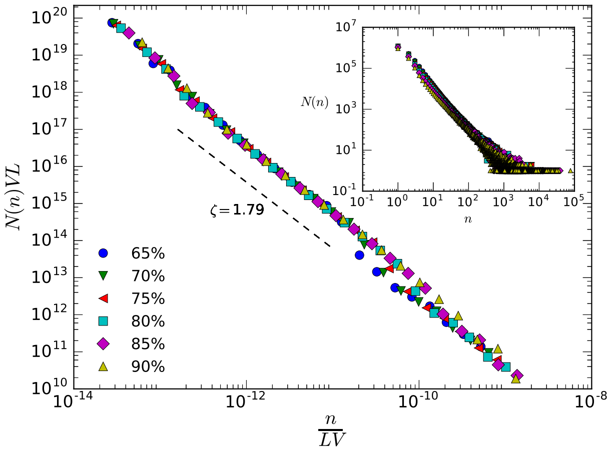

(e) Spanish language

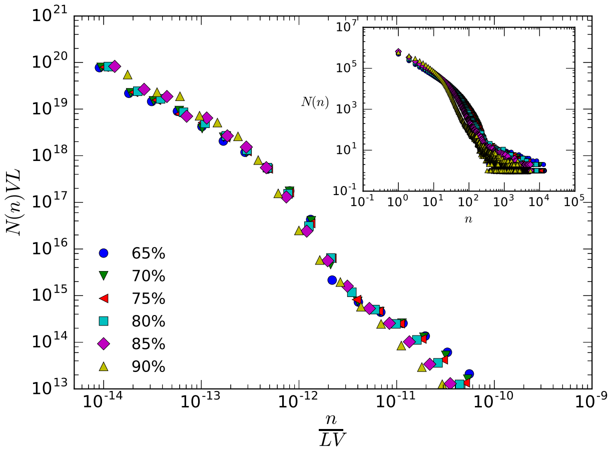

(f) Basque null model

Figure 6: **Zipf's law, KALAKA database.** Log-log plot of Zipf's law for several thresholds θ, from a to e each panel corresponds to one different language of Kalaka speech database. The inset panel shows the raw, threshold-dependent distributions and the outer panel Zipf's law has been collapsed and logarithm binning applied to improve the statistics at the tail of the distribution. Last panel corresponds to the spanish null model where energy release distribution is not fulfilled (and the distributions do not collapse).

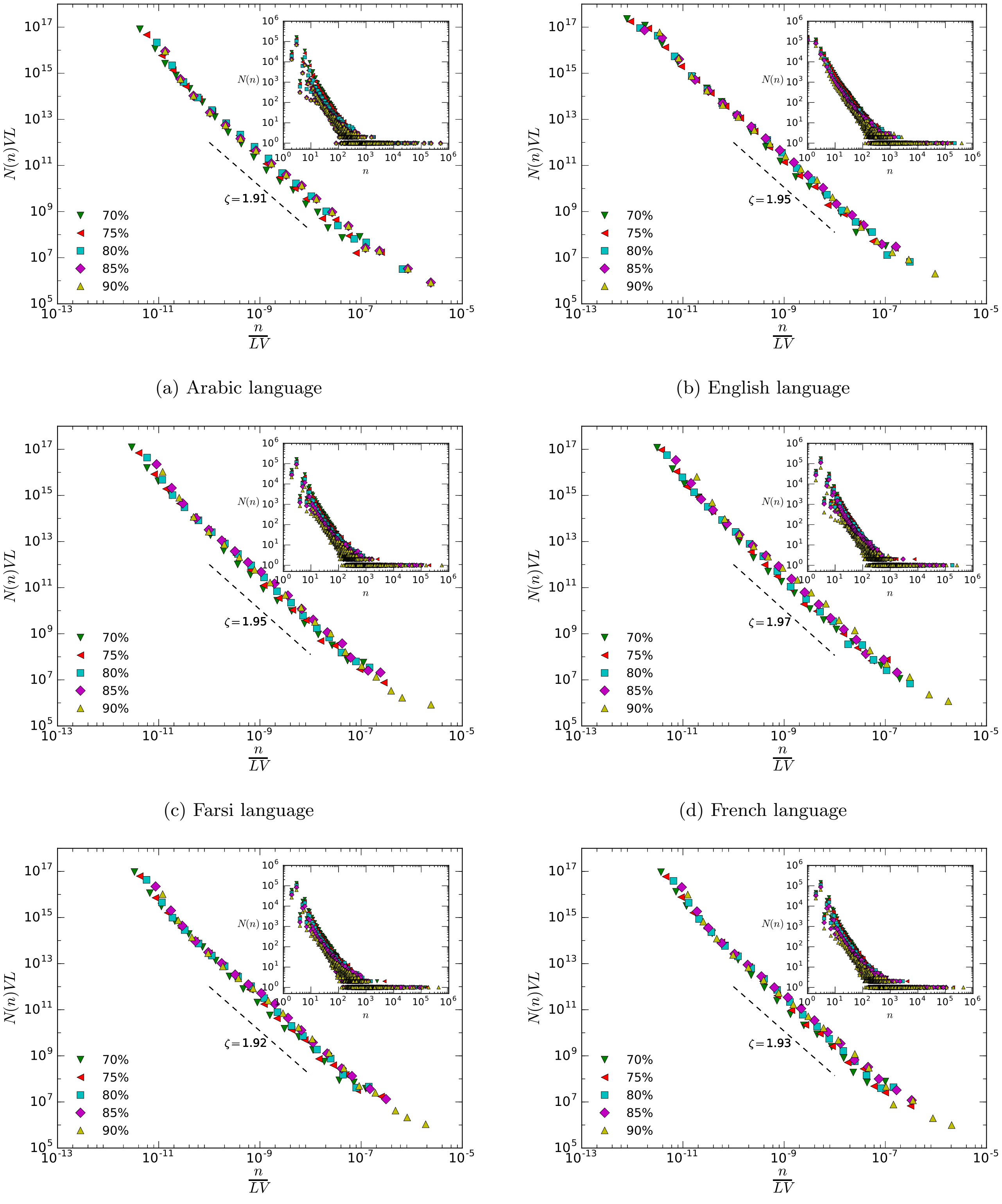

(a) Arabic language

(b) English language

(c) Farsi language

(d) French language

(e) German language

(f) Hindi language

Figure 7: **Zipf's law, LRE database.** Log-log plot of Zipf law for several thresholds θ and languages from LRE speech database (see Figure 8 for more languages of this dataset). The inset panel shows the raw, threshold-dependent distributions and the outer panel Zipf's law has been collapsed and logarithm binning applied for improving the tail of the distribution.

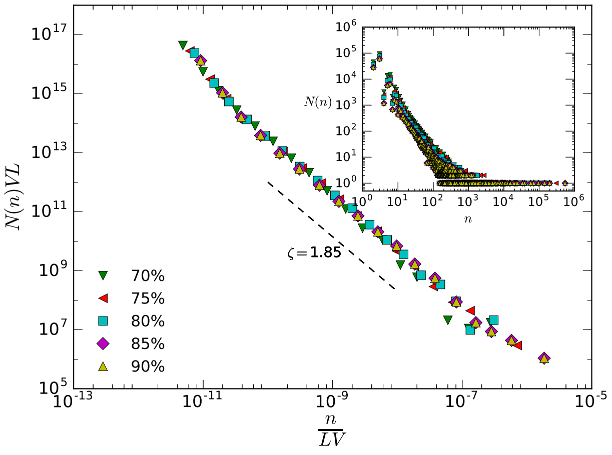

(a) Japanese language

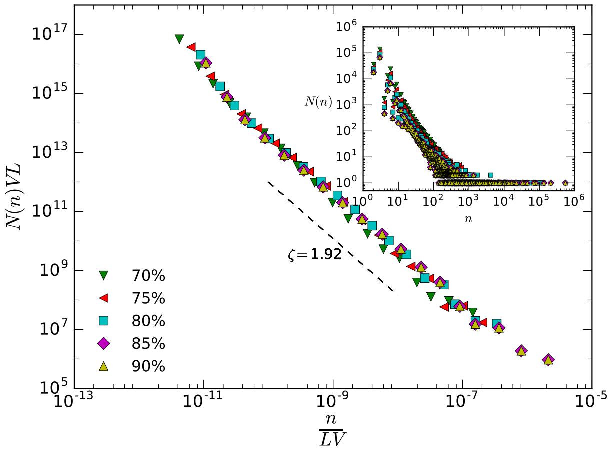

(b) Korean language

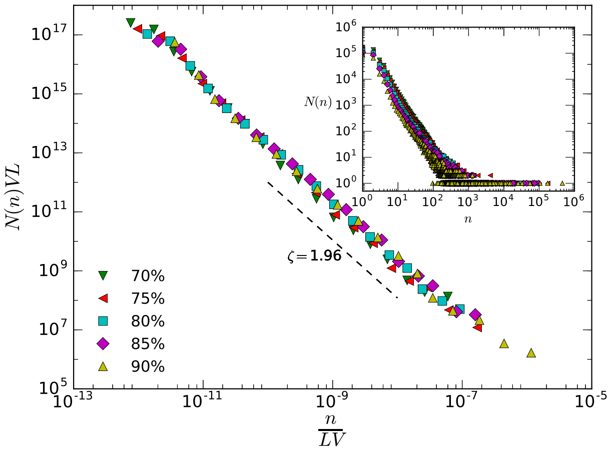

(c) Mandarin language

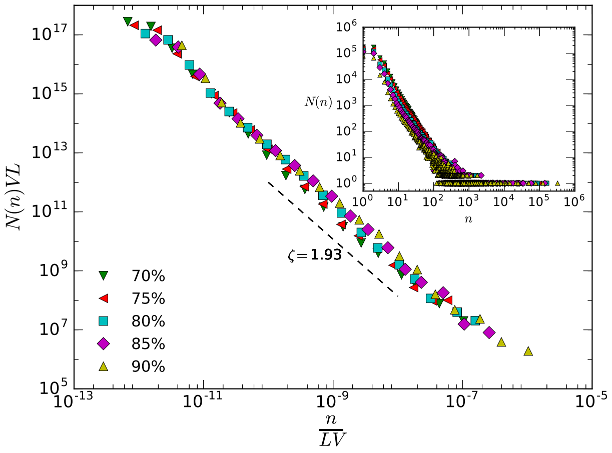

(d) Spanish language

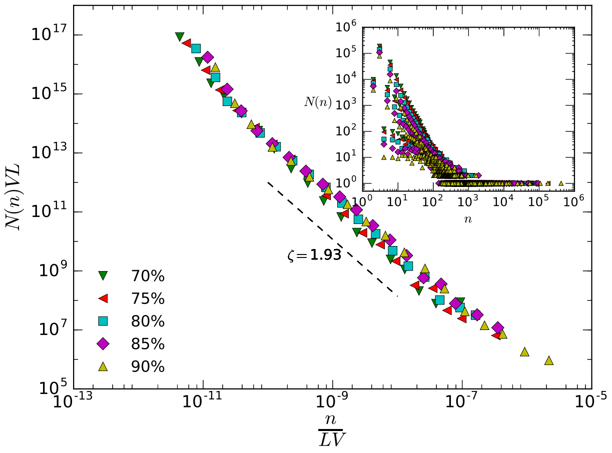

(e) Tamil language

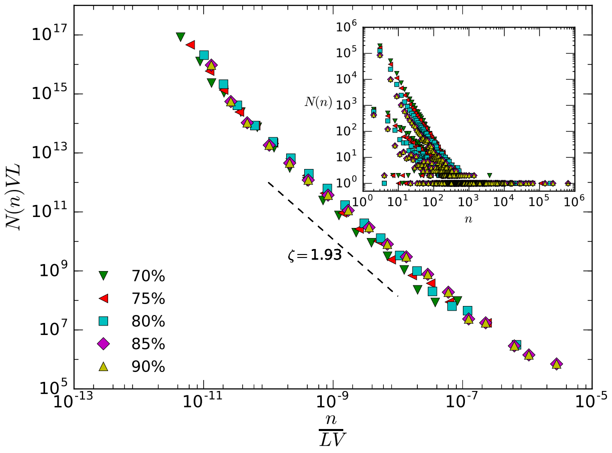

(f) Vietnamese language

Figure 8: **Zipf's law, LRE database (continued).** Log-log plot of Zipf law for several thresholds θ and languages extracted from the LRE speech database (see Figure 7 for more languages of this dataset). The inset panel shows the raw, threshold-dependent distributions and the outer panel Zipf's law has been collapsed and logarithm binning applied for improving the tail of the distribution.

8 Heaps' law: additional figures

In this section we plot the Heaps laws for all languages considered in this study. We recall that the relation between the original (α) and the collapsed exponent (α') is $\alpha' = \alpha/(1+\alpha)$. Results are qualitatively similar across languages and exponents are compatible with a language-independent process. We also report the analogous law obtained from the null model of the portuguese database, consisting of reshufling the instantaneous energy signal $\epsilon(t)$, for which we find the trivial law with an exponent close to 1 ($\alpha = 0.95$). (Note again that as the KALAKA database is larger than the LRE database, the curves for the corresponding languages associated to the KALAKA case are smoother and the fitting exponents have less error than the ones associated to the LRE database).

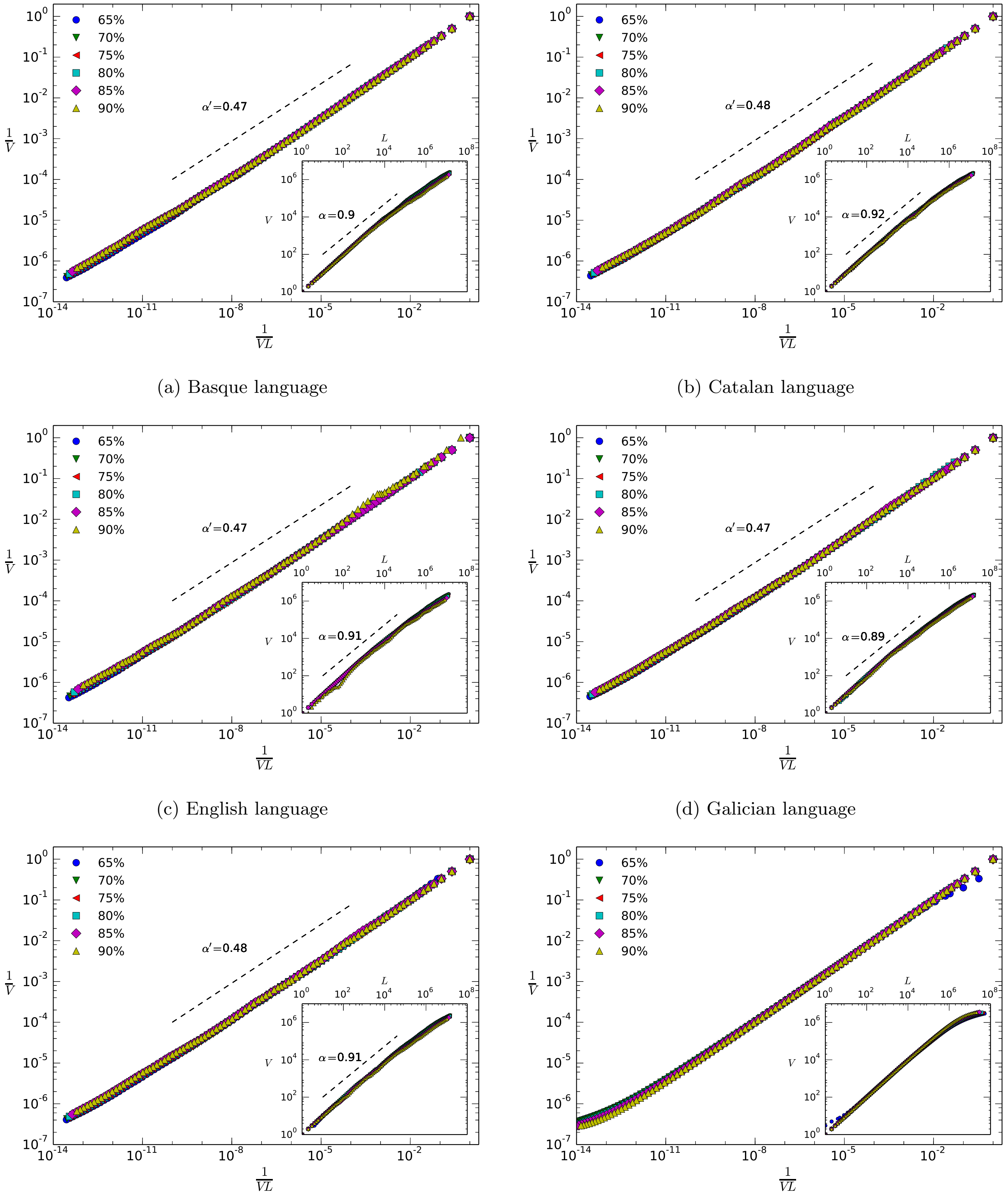

(a) Basque language (b) Catalan language

(c) English language (d) Galician language

(e) Spanish language (f) Portuguese null model

Figure 9: **Heaps's law, KALAKA database.** Log-log plot of Heap's law for several thresholds θ, for panels a to e each subfigure corresponds to one different language of Kalaka speech database. In the inner panel we show how the number of different tokens (V) increases sublinearly with the size of the series (L), where the slope can be estimated properly for about three decades. In the main panel we show the collapses. Last subfigure corresponds null model, plotting Heap's law for randomized signal extracted from the Portuguese dataset. The scaling saturates to the trivial law with α close to 1.

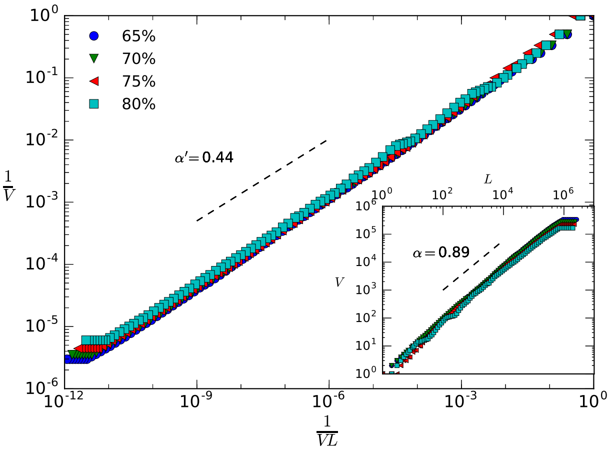

(a) Arabic language

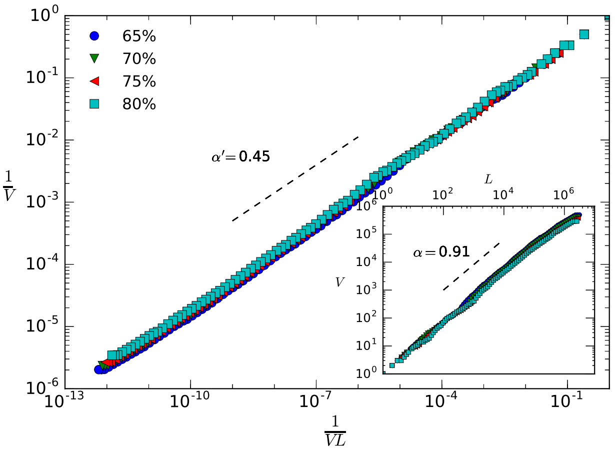

(b) English language

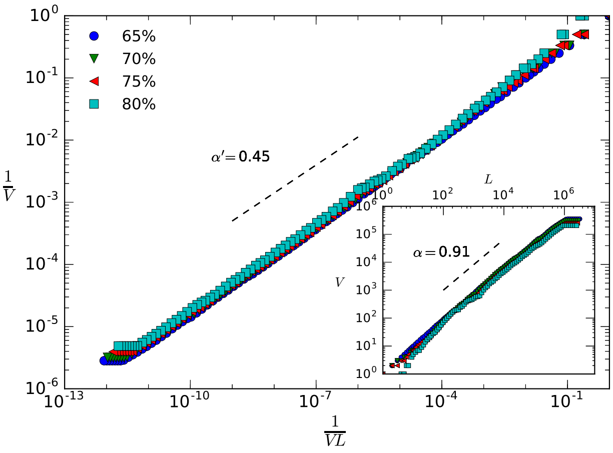

(c) Farsi language

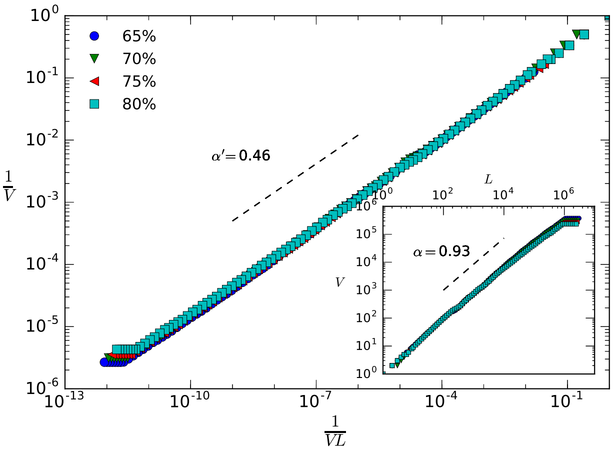

(d) French language

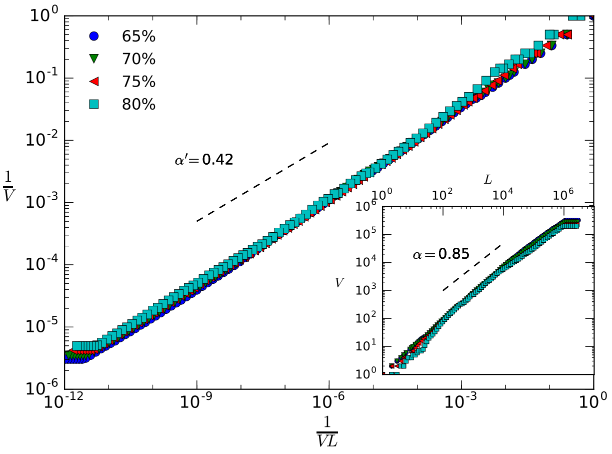

(e) German language

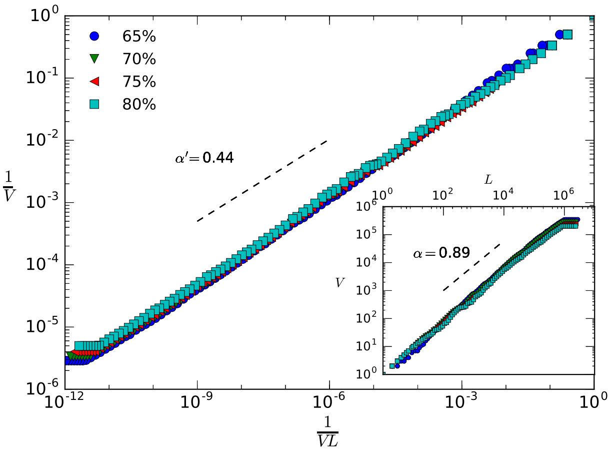

(f) Hindi language

Figure 10: **Heaps's law, LRE database.** Log-log plot of Heaps's law for several thresholds θ and languages of LRE speech database. In the inner panel we show how the number of different tokens (V) increases sublinearly with the size of the series (L), where the slope can be estimated properly for about three decades. In the main panel we show the collapses.

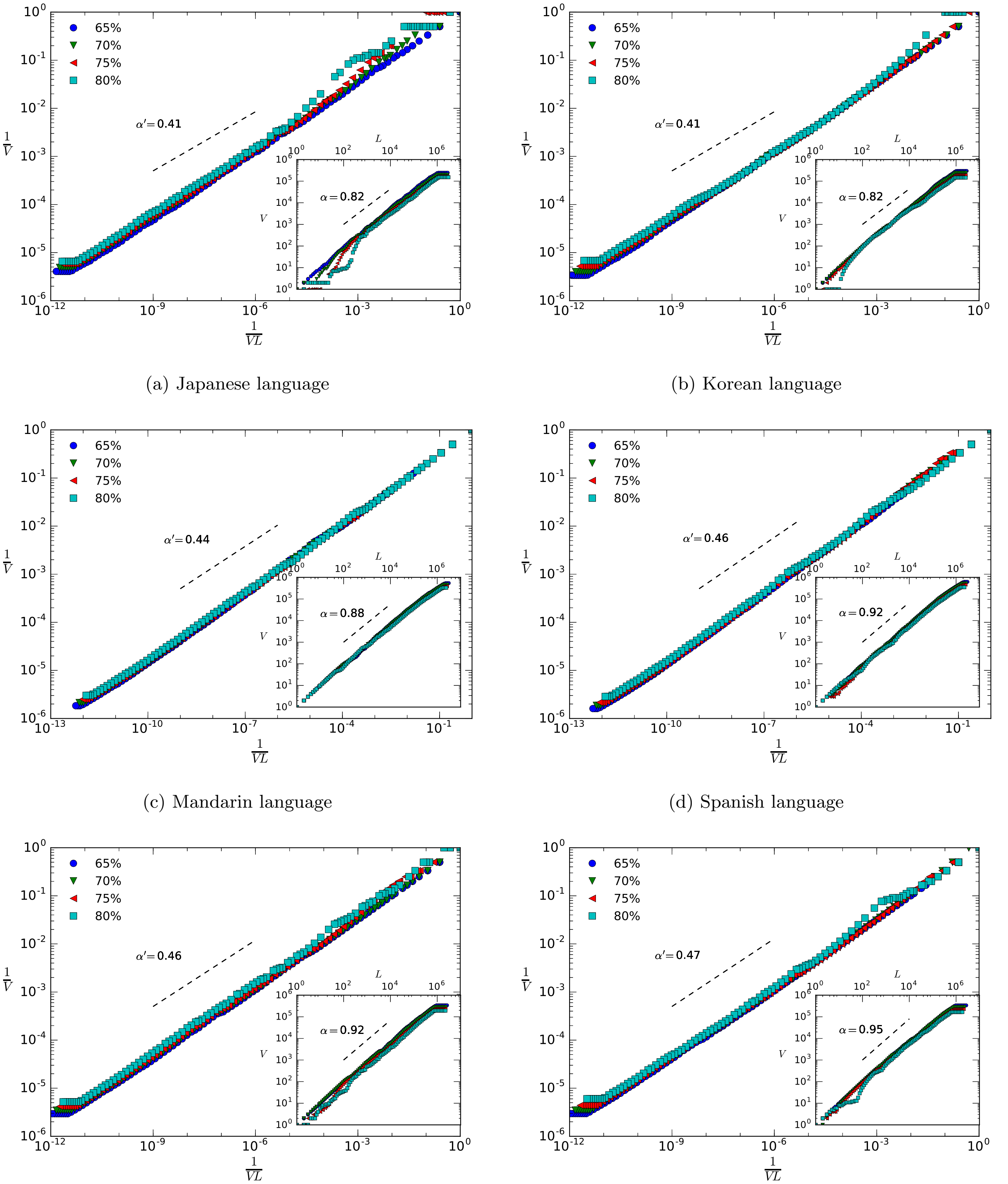

(a) Japanese language

(b) Korean language

(c) Mandarin language

(d) Spanish language

(e) Tamil language

(f) Vietnamese language

Figure 11: **Heaps's law, LRE database (continued).** Log-log plot of Heaps's law for several thresholds θ and languages of LRE speech database. In the inner panel we show how the number of different tokens (V) increases sublinearly with the size of the series (L), where the slope can be estimated properly for about three decades. In the main panel we show the collapses.

9 Brevity law: additional figures

Note that already the temporal distribution of voice events -describing the frequency of appearance of tokens with a certain duration- could be a direct observation of the brevity law. This distribution is plotted in log-log for all languages and $\theta = 85\%$ in figure 12. The simple observation that these distributions are monotonically decreasing functions is already a strong support of the brevity law, as these point out that short tokens are more frequent than those that last longer.
In this section we also plot the Brevity laws for all languages considered in this study. Results are qualitatively similar across languages and exponents are compatible with a language-independent process. We also report the analogous law obtained from the null model of the english database, consisting of reshufling the instantaneous energy signal $\epsilon(t)$. We don't observe a good collapse in this latter case (results are equivalent for the null models generated for all other languages). (Note again that as the KALAKA database is larger than the LRE database, the curves for the corresponding languages associated to the KALAKA case are smoother and the fitting exponents have less error than the ones associated to the LRE database).

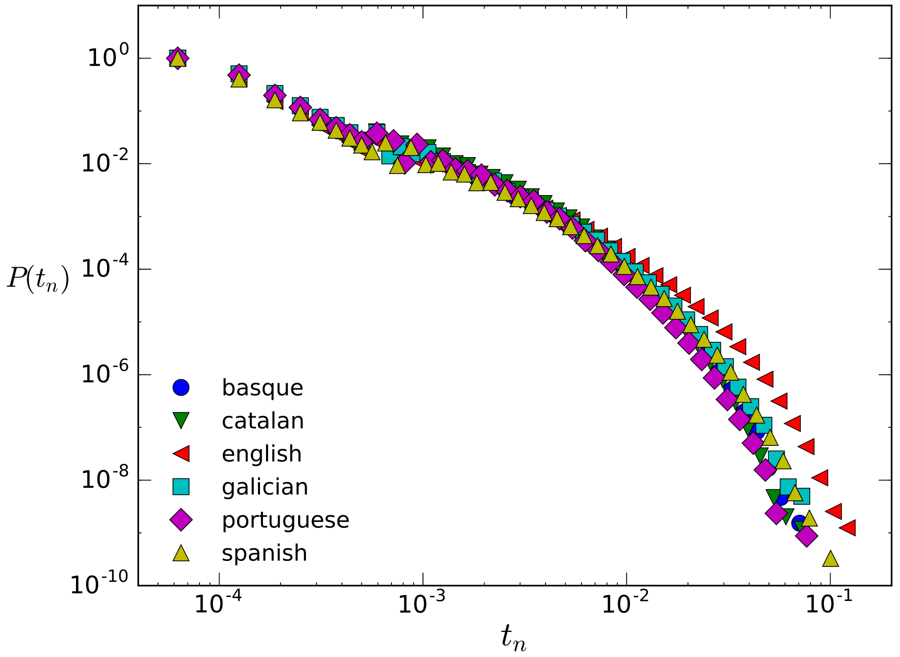

Figure 12: Voice events duration distribution for several languages and using a threshold of $\theta = 85$ in the KALAKA database

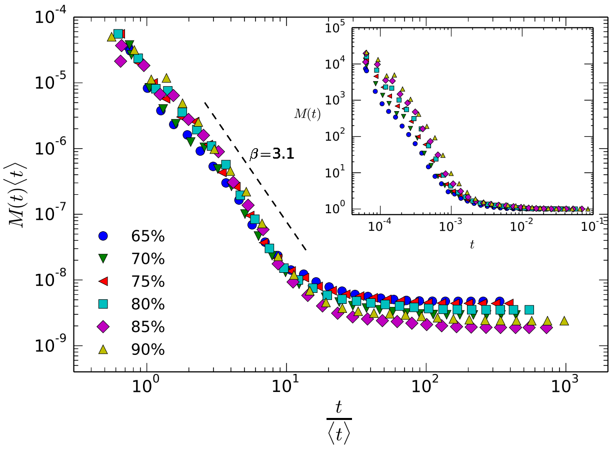

(a) Basque language

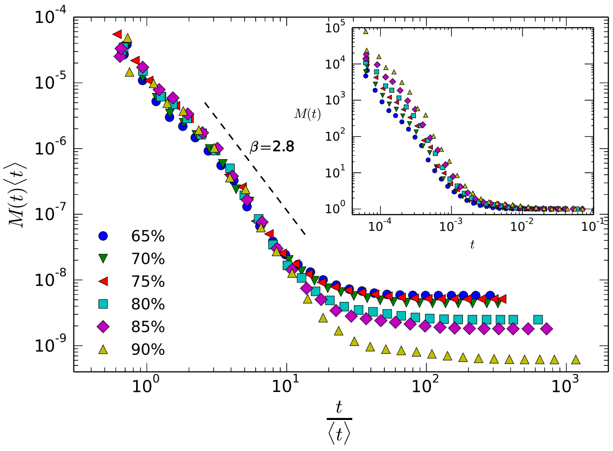

(b) Catalan language

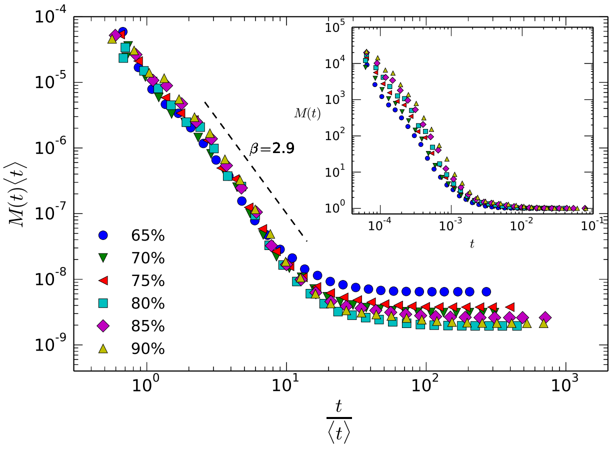

(c) Galician language

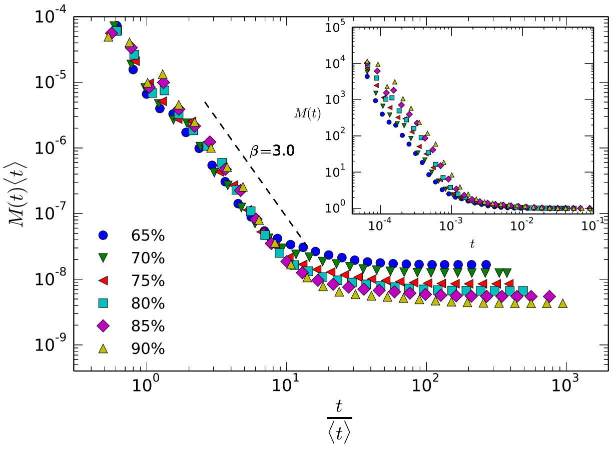

(d) Portuguese language

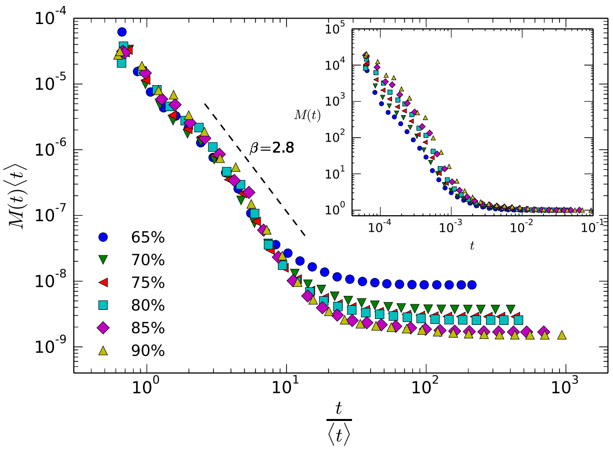

(e) Spanish language

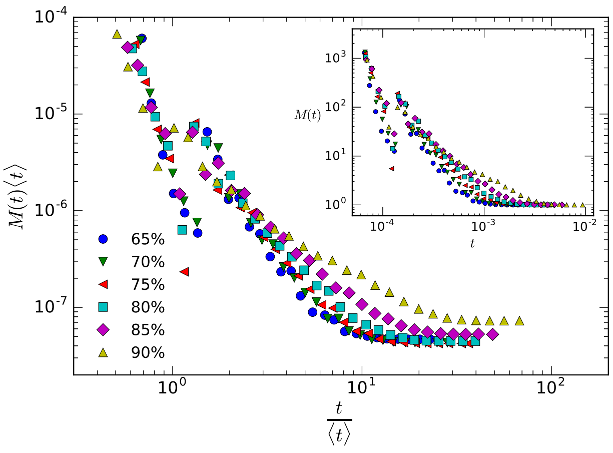

(f) English null model

Figure 13: **Brevity law, KALAKA database.** Log-log plot of the Brevity law for several thresholds and languages belonging to Kalaka database (from a to e. After computing the vocabulary of the signal, the mean duration of each different type is calculated. In the inner panel it is shown the frequency of the vocabulary M(t) depending on its duration t for each θ after a logarithmic binning to reduce the dispersion. In the outer panel, the duration of voice events is collapsed with the mean duration showing the independence of the scaling parameter from the θ. Figure f shows null model, plotting Brevity Law for the English dataset. In this case, brevity law is not fulfilled (and the distributions don't collapse).

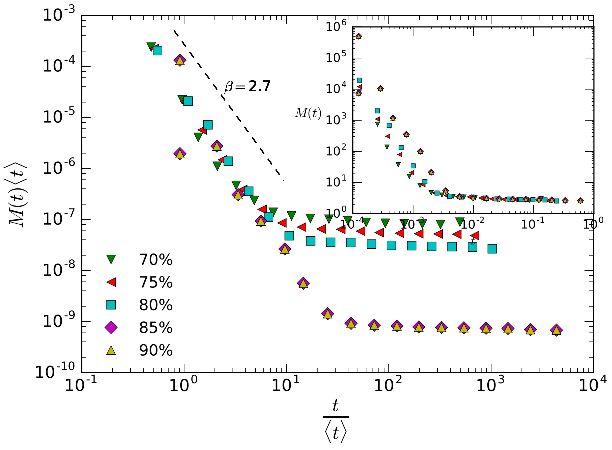

(a) Arabic language

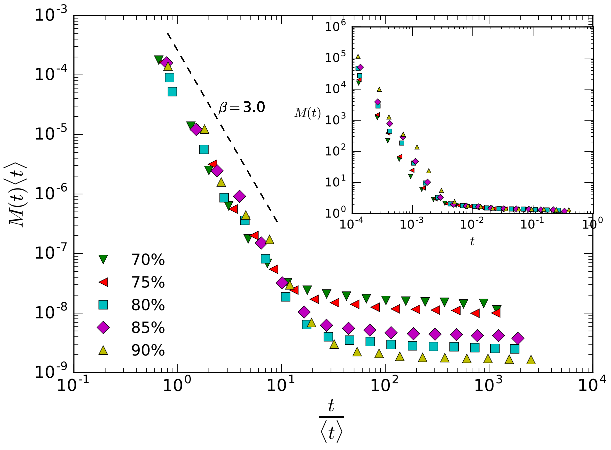

(b) English language

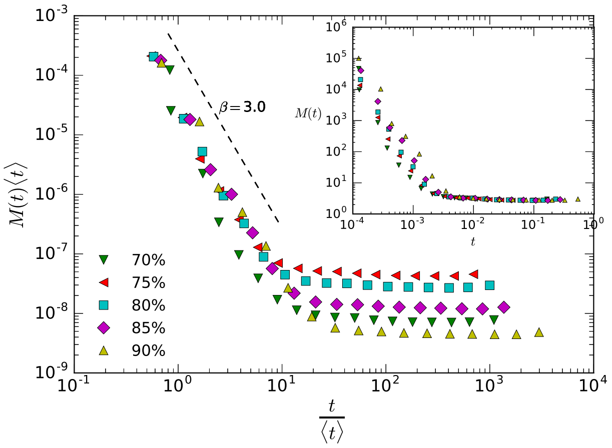

(c) Farsi language

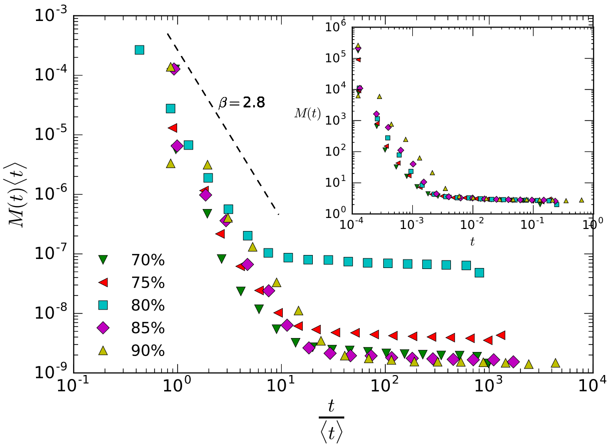

(d) French language

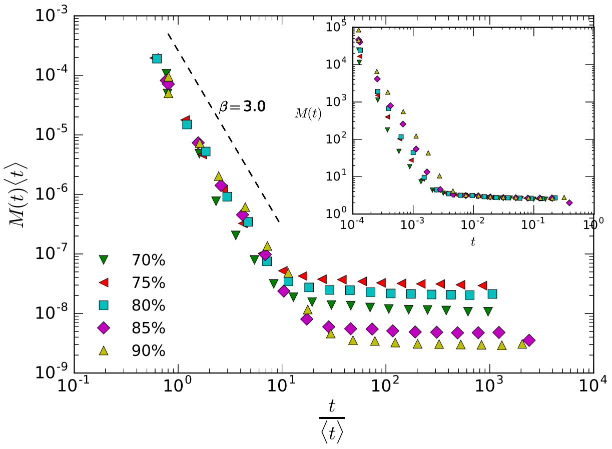

(e) German language

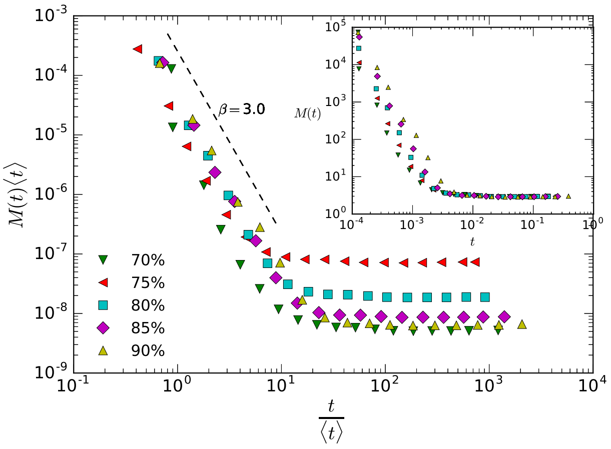

(f) Hindi language

Figure 14: **Brevity law, LRE database.** Log-log plot of the Brevity law for several thresholds and languages belonging to Kalaka database (from a to f: after computing the vocabulary of the signal, the mean duration of each different type is calculated. In the inner panel we show the frequency of the vocabulary M(t) depending on its duration t for each θ after a logarithmic binning to reduce the dispersion. In the outer panel, the duration of voice events is collapsed with the mean duration showing the independence of the scaling parameter from the θ.

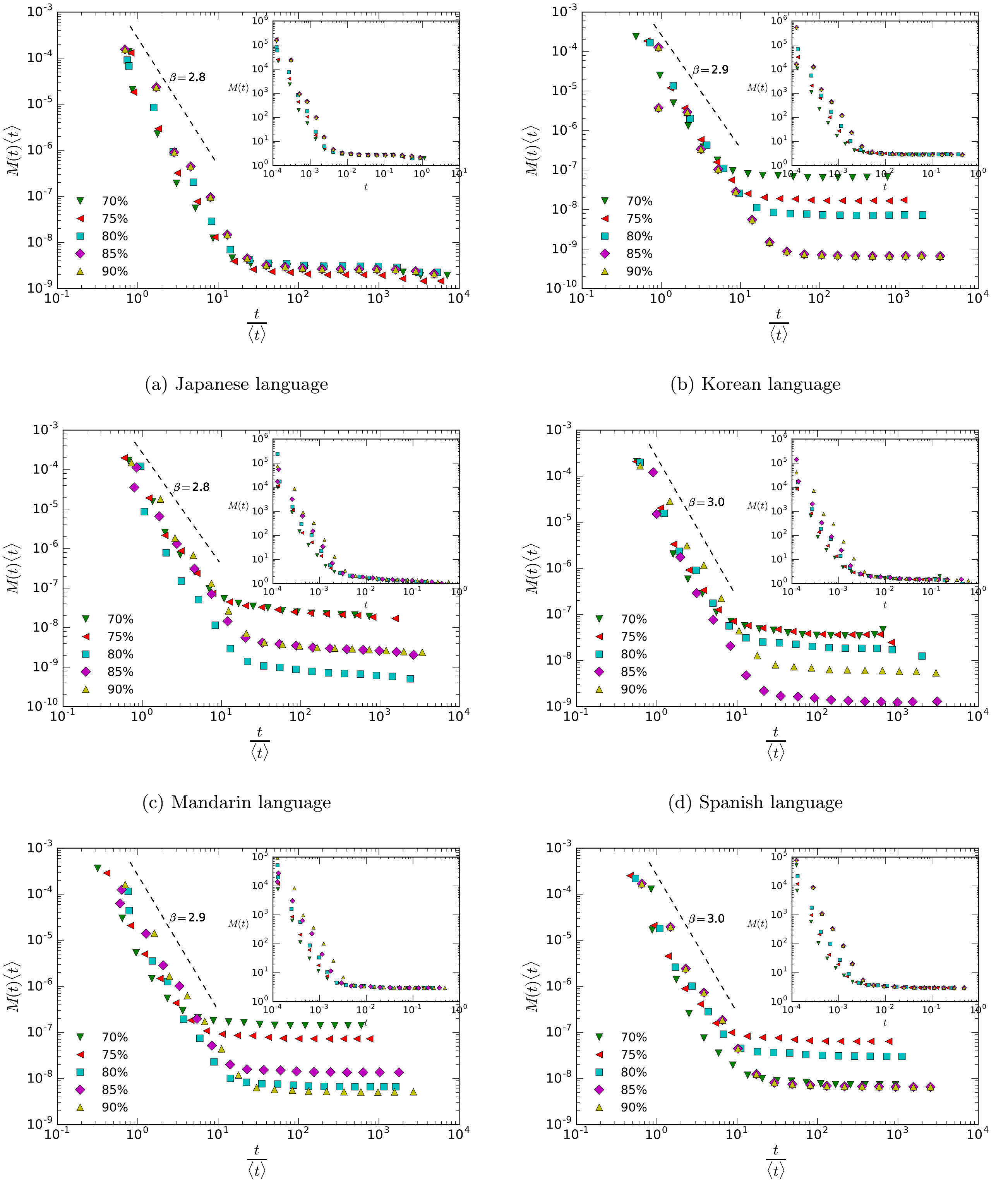

(a) Japanese language

(b) Korean language

(c) Mandarin language

(d) Spanish language

(e) Tamil language

(f) Vietnamese language

Figure 15: **Brevity law, LRE database (continued).** Log-log plot of the Brevity law for several thresholds and languages belonging to Kalaka database (from a to f: after computing the vocabulary of the signal, the mean duration of each different type is calculated. In the inner panel we show the frequency of the vocabulary M(t) depending on its duration t for each θ after a logarithmic binning to reduce the dispersion. In the outer panel, the duration of voice events is collapsed with the mean duration showing the independence of the scaling parameter from the θ.

References

[1] H. Brehm and W. Stammler. Description and generation of spherically invariant speech-model signals. *Signal Processing*, 12(2):119–141, 1987.

[2] W. B. Davenport Jr. An experimental study of speech-wave probability distributions. *The Journal of the Acoustical Society of America*, 24(4):390–399, 1952.

[3] S. Gazor and W. Zhang. Speech probability distribution. *Signal Processing Letters, IEEE*, 10(7):204–207, 2003.

[4] M. Paez and T. Glisson. Minimum mean-squared-error quantization in speech PCM and DPCM systems. *IEEE Transactions on Communications*, 20(2):225–230, apr 1972.

[5] D. Richards. Statistical properties of speech signals. In *Proceedings of the Institution of Electrical Engineers*, volume 111, pages 941–949. IET, 1964.
